# Handheld versus mounted laser speckle contrast perfusion imaging demonstrated in psoriasis lesions

**DOI:** 10.1038/s41598-021-96218-6

**Published:** 2021-08-17

**Authors:** Ata Chizari, Mirjam J. Schaap, Tom Knop, Yoeri E. Boink, Marieke M. B. Seyger, Wiendelt Steenbergen

**Affiliations:** 1 Biomedical Photonic Imaging, Technical Medical Centre, Faculty of Science and Technology, University of Twente, P.O. Box 217, 7500 AE Enschede, The Netherlands; 2 Department of Dermatology, Radboud University Medical Center, P.O. Box 9101, 6500 HB Nijmegen, The Netherlands; 3 Multi-Modality Medical Imaging, Technical Medical Centre, Faculty of Science and Technology, University of Twente, P.O. Box 217, 7500 AE Enschede, The Netherlands; 4 Department of Applied Mathematics, University of Twente, P.O. Box 217, 7500 AE Enschede, The Netherlands

**Keywords:** Biomedical engineering, Imaging and sensing, Translational research, Optical imaging

## Abstract

Enabling handheld perfusion imaging would drastically improve the feasibility of perfusion imaging in clinical practice. Therefore, we examine the performance of handheld laser speckle contrast imaging (LSCI) measurements compared to mounted measurements, demonstrated in psoriatic skin. A pipeline is introduced to process, analyze and compare data of 11 measurement pairs (mounted-handheld LSCI modes) operated on 5 patients and various skin locations. The on-surface speeds (i.e. speed of light beam movements on the surface) are quantified employing mean separation (MS) segmentation and enhanced correlation coefficient maximization (ECC). The average on-surface speeds are found to be 8.5 times greater in handheld mode compared to mounted mode. Frame alignment sharpens temporally averaged perfusion maps, especially in the handheld case. The results show that after proper post-processing, the handheld measurements are in agreement with the corresponding mounted measurements on a visual basis. The absolute movement-induced difference between mounted-handheld pairs after the background correction is $16.4\pm 9.3~\%$ (mean ± std, $n=11$), with an absolute median difference of $23.8\%$. Realization of handheld LSCI facilitates measurements on a wide range of skin areas bringing more convenience for both patients and medical staff.

## Introduction

The microcirculation in the skin is responsible for delivering oxygen and nutrients to the tissue and evaluation of the microvasculature provides insights regarding tissue viability and health. Microcirculatory perfusion imaging has the potential to provide valuable information on the microvasculature and has gained attention in the past years^1–4^. Examples of medical applications of perfusion imaging include psoriasis^5–7^, burn wound depth assessment^8^ and postoperative monitoring^9^. Among the optical perfusion imaging techniques, laser speckle contrast imaging (LSCI) has been a point of interest in the past three decades since it is non-invasive, affordable and compact^10^. In this imaging modality, the skin surface is illuminated by coherent laser light. Due to the roughness of the surface and multiple light scattering inside the tissue, an interference pattern is observed on the camera detector called optical speckle. Blurred speckle patterns are created by movements of red blood cells (RBCs) within a certain exposure time. Their contrast depends on the movement: a larger movement causes lower speckle contrast^11^. The speckle contrast distribution can be transformed into a perfusion map of the illuminated tissue.

The sensitivity of optical speckles to movements of other sources, such as involuntarily movements of the patients, introduces challenges during (handheld) measurements^12^. Several attempts to reduce the effect of these movements have been described in literature. Using an adjacent opaque surface, Mahe et al.^13^ obtained mounted LSCI data over a moving skin surface as well as during exercise^14^ and compensated the occurred movement artefacts. Their protocol was later optimized by Omarjee et al.^15^. Due to the need to make the LSCI system portable, which is especially important in a clinical setting, Lertsakdadet et al.^16^ proposed a handheld LSCI device with a fiducial marker as an indicator of movement artefacts as well as introducing a motion stabilized version of this device using a motorized gimbal mount^17^. To date, all approaches are based on attachment of an opaque surface on the tissue for frame alignment and accounting for movement artefacts. The room for improvement is to find alternatives for such opaque surfaces so that they can be removed.

In a previous work, we measured movements of the LSCI system during handheld experiments using an electromagnetic (EM) tracker. We analyzed movement artefacts in response to on-surface beam speed, that is the speed of the light beam on the surface caused by translation and rotation of the probe, and wavefront tilting for media with various scattering levels^18^. In addition, we explored the influence of wavefront types on movement artefacts employing three types of illumination, namely scrambled, spherical and planar wavefronts^19^. Results of handheld measurements showed that on average spherical and planar wavefronts cause less drop in the speckle contrast compared to a scrambled wavefront while measuring on a tissue mimicking static phantom.

In this work, we explore the validity of handheld measurements compared to mounted measurements (as the golden standard) demonstrated in psoriatic skin. We chose to use psoriasis lesions since it is well known that microvasculature changes and angiogenesis are important in psoriasis^20,21^. Additionally, perfusion inhomogeneity is present in psoriasis plaques. This results in so called hot spots and cold spots^5,22^, that provides valuable perfusion maps for comparison of mounted and handheld measurements. A methodology is proposed to post-process the acquired raw speckle frames under the presence of small natural movements of patients and operators, and to compute a representative perfusion map per experiment. Moreover, the theoretical model for assigning perfusion values to the measured speckle contrast is examined in-vitro and in-vivo. The aim is to study the proportionality of estimated perfusion and the applied speed.

## Methods

### Handheld perfusion imager (HAPI)

**Figure 1:**
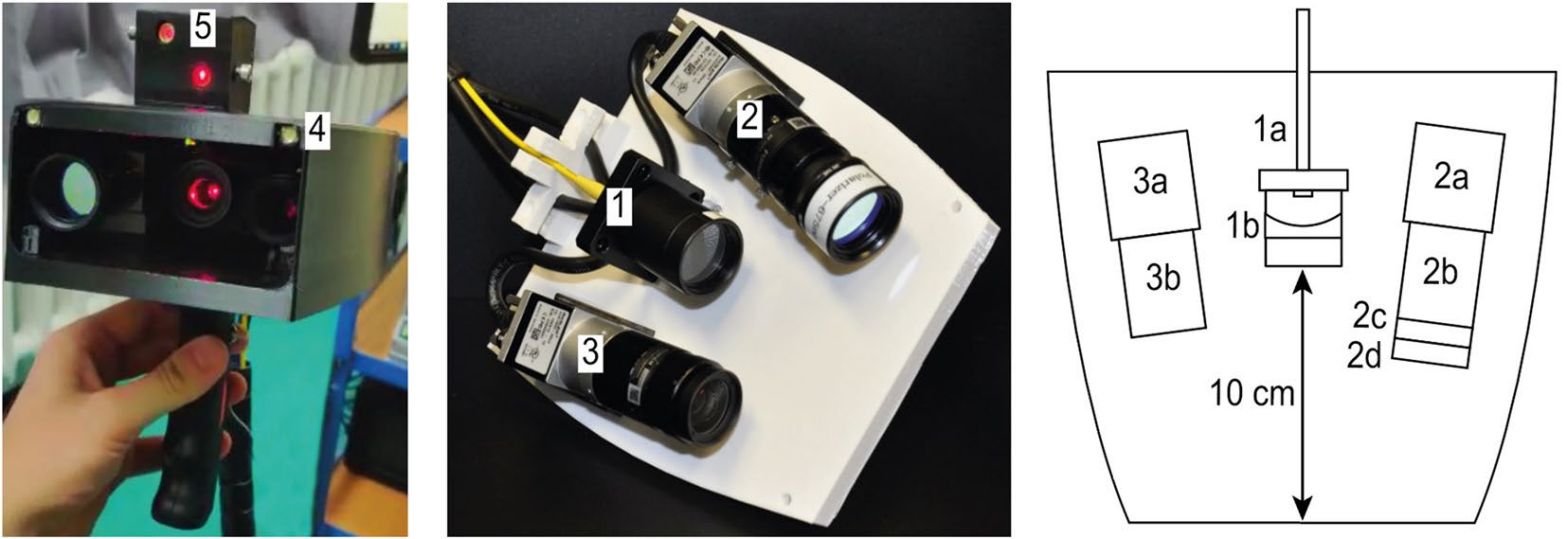
Experimental setup of the handheld perfusion imager (HAPI). Left, a front view of the handheld probe. Middle, handheld probe without a cap (reprinted from^18^). Right, schematic drawing of the handheld probe. 1, Fiber tip (1a) and lens for laser beam expansion (1b). 2, Set of monochromatic camera (2a), objective lens (2b), bandpass filter (2c) and linear polarizer (2d). 3, Set of RGB camera (3a) and objective lens (3b). 4, Power light emitting diodes (LEDs) for white-light illumination. 5, Targeting lasers. The total weight of the handheld probe is $995\pm 25$ g.

A handheld LSCI device was designed to be utilized in a clinical research setting, performing both red-green-blue (RGB) and perfusion imaging. Figure 1 illustrates an overview of the experimental setup.

For perfusion imaging, a coherent and continuous wave single longitudinal mode laser (CNI MSL-FN-671) with a wavelength of 671 nm and output power of 300 mW was used with a coherence length $>50$ m. The laser beam was attenuated by an absorptive filter of 0.2 optical density (Thorlabs NE02A) with a non-perpendicular surface to the beam, preventing direct back-reflection into the laser. The laser beam was then directed to an olympus plan achromat microscope objective (Thorlabs, RMS10X), that was mounted on a three axis stage (Thorlabs Nanomax 300), via broadband dielectric mirrors (Thorlabs BB1-E02). The three axis stage was used to focus the light into a single mode optical fiber (Thorlabs P1-630A-FC-5) and an FC/APC connection to prevent back-reflections to the laser head. The distal end of the optical fiber is attached to the handheld probe where its outgoing beam has been diverged using a mounted plano-concave lens (Qioptiq, N-BK 7) of focal distance $-6$ mm, diameter of 6 mm and was located 25 mm away from the fiber tip. This leads to a diverging laser beam with a spherical wavefront. The distance from the light source and camera sensors to the tissue surface has been set to 40 cm, although it varied slightly during handheld operations. The measured beam width, i.e. the radial distance at which the intensity decreases by a factor of $1/e^2$ of its maximum at the center of illumination, for this system is approximately 8 cm. Also, the full width at half max (FWHM) of the beam intensity is measured as 8.8 cm. Previously, we demonstrated that, using a spherical wavefront, movement artefacts due to the rotation of the probe could be reduced in comparison to the use of a conventional engineered diffuser which generates a scrambled wavefront^19^.

To ensure eye safety, the following precautions are taken: (1) use of a visible wavelength (i.e. red) compared to near infrared; (2) training of the operator of the handheld probe prior to the study. To allow or block laser illumination on the tissue, a motorized shutter was made by a translational stage (Zaber, T-LSM050A). Two cross-line laser modules with optical power 5 mW and operating wavelength 650 nm, each driven by 25 mA electrical current, were used to illuminate the boundaries of the imaging fields-of-views (FOVs). This aided the operator in targeting the imaging regions during perfusion imaging. The RGB camera was not of help for targeting the imaging regions during perfusion measurement, because the white light illumination was switched off.

To record the speckle intensity patterns for the patient measurements, a monochrome camera (Basler acA2040 55um USB3) was used that operated at a frame rate of 30 Hz, imaging depth of 8 bits, gain of 10 dB, exposure time of 10 ms and frame size of 1536 px $\times$ 2048 px. The camera objective (FUJINON HF16XA-5M) had a focal length of 16 mm and an f-number of *F*/8 in order to obtain the optimum point for (1) the detected light intensity to have the highest dynamic range for computation of speckle contrast and (2) the speckle size to meet the Nyquist criterion^23^. The magnification of the imaging system was measured as 11.9 px/mm resulting in an FOV of $12.9~{\mathrm{cm}}\times 17.2~{\mathrm{cm}}$. To reduce noise from background light, a hard coated bandpass interference filter (Edmund Optics) of wavelength $675\pm 12.5$ nm was mounted on the objective as well as turning off the general illumination of the room during each measurement. A linear polarizer (Thorlabs LPNIRE 100-B) with a polarization direction perpendicular to that of the laser beam was used in the imaging system to minimize specular reflections and to increase measurable speckle contrast.

For making RGB images, a color camera (Basler acA1920-40uc USB3) was used which operated at the imaging depth of 8 bits, gain of 12 dB, exposure time of 10 ms and frame size of $1200~{\mathrm{px}}\times 1920$ px. Its objective (FUJINON HF12XA-5M) had a 12 mm focal length and worked with the fully open diaphragm. With a magnification of 5.6 px/mm the obtained FOV was $21.4~{\mathrm{cm}}\times 34.3~{\mathrm{cm}}$. Two power light emitting diodes (LEDs) of each 1 W maximum electrical power and driven by 90 mA electrical current were used as illumination sources during the imaging.

### Study population

Five adult psoriasis patients (2 female and 3 male) participated in this study. In total, eleven pairs of mounted and handheld measurements were carried out. The time interval between each measurement pair was minimized to maximally 9 minutes (Supplementary Tables S1–11). In order to minimize external influences on the skin perfusion level, patients were asked to refrain from heavy physical activities, scratching of the skin, drinking caffeine or smoking for at least 30 min prior to each measurement. The in-vivo evaluation of the perfusion estimation model mentioned in Results *Contrast-perfusion model examination* was performed on a healthy volunteer.

#### Ethics declarations

Informed consent was obtained from all participants before enrollment. Utilization of the HAPI and the study protocol was approved by the ethics committee of the region of Arnhem-Nijmegen and the Radboud university medical center, Nijmegen, the Netherlands (NL69174.091.19). All experiments were performed in accordance with relevant guidelines and regulations.

### Measurement protocol

The laser source for perfusion imaging was turned on at least 15 min prior to each measurement to warm up. A calibration cap was placed in front of the handheld probe to take a snapshot of a Delrin plate (Polyoxymethylene) and a scattering suspension (Perimed, PF 1001 Refill Motility Standard) located at its working distance. The PF 1001 Refill Motility Standard is a colloidal suspension of polystyrene particles. For a particle diameter of 320 nm, the concentration of the suspension would be $1.2\times 10^{12}$ per cube centimeter^24^. The average speckle intensity on the Delrin plate was checked via a custom-made user-interface in MATLAB R2019b (Mathworks)^25^ and maximized by manually adjusting the three axis stage on which the microscope objective directs laser light into the optical fiber. The obtained average intensities ${\bar{I}}$ are between 13.5–21.9 out of 255. The eye safety and the maximum tolerable optical power through a single mode optical fiber are two limiting factors in obtaining a higher average intensity level in the imaging plane. The camera gain was used to enhance the detected intensity level. Use of a bandpass filter resulted in a high signal-to-noise-ratio (SNR) which facilitated detection of suggestive heartbeat patterns by monitoring temporal fluctuations of spatial speckle contrast on a lesion. Moreover, temporal averaging of the perfusion maps ensured obtaining a clear difference between areas with low and high perfusion values. The speckle contrasts of regions selected on the Delrin plate and scattering suspension were in the ranges of $C_{\mathrm{s}}=0.64-0.94$ and $C_{\mathrm{d}}=0.16-0.24$, respectively (Supplementary Tables S1–11). This calibration data was not used for adjusting data but for checking the stability of the system.

Black marker dots (Edding 400, 1 mm, permanent marker) were placed by a physician on the clinical psoriatic lesion border to provide a reference of the clinically visible lesion borders on the perfusion images. Depending on the skin area to be imaged, subjects were located in a rest position: sitting or supine. Subjects were asked to stay still and breath normally during the measurements. Measurements started with capturing an RGB frame. During the RGB imaging, the LEDs were switched on and the laser illumination was blocked via the motorized shutter. After the RGB imaging, the LEDs were turned off while the laser illumination was allowed on the subject and the monochrome camera acquired speckle frames for 7 s. All of the experiments were performed by the same operator , in the same room, with the curtains closed and room illumination switched off. The operator was instructed to perform the handheld measurements while conveniently positioned, the arm bent at almost $90^{\circ }$ and keeping the handheld probe normally (i.e. without over-concentration). For the mounted measurements, the handheld LSCI device was placed on a tripod.

### Data analysis

**Figure 2:**
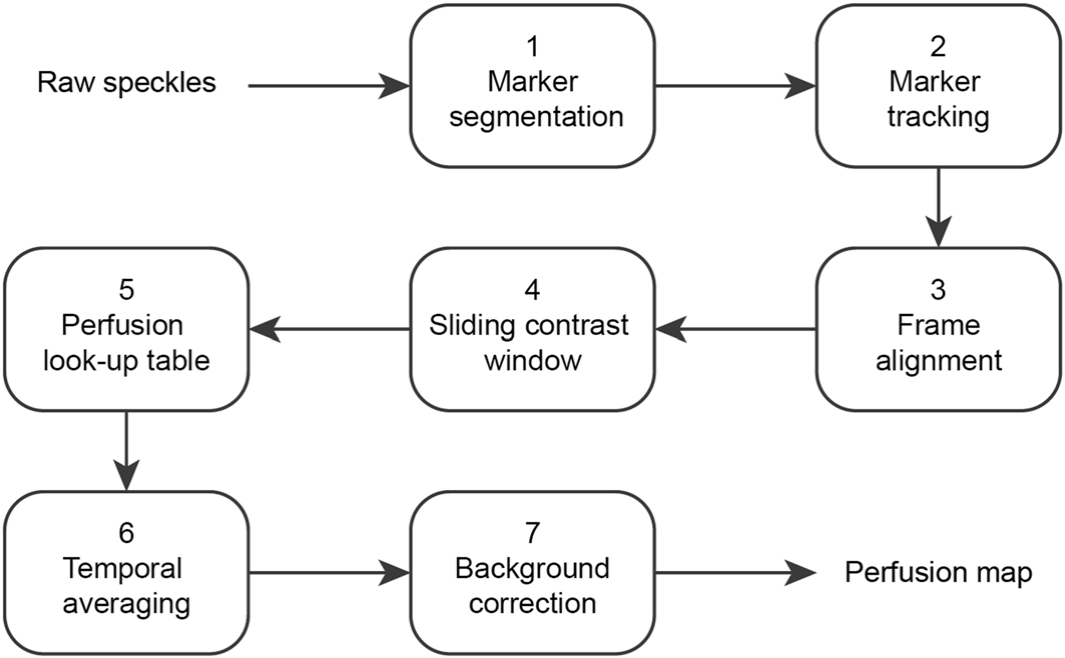
Analysis workflow for perfusion imaging. (1) Choosing an area within the speckle frames that includes markers and run the segmentation algorithm^26,27^. (2) Localization of segmented markers for the entire stack of frames and calculation of horizontal and vertical on-surface speed elements. (3) Translating the frames based on the computed two dimensional displacements with respect to the reference frame. (4) Applying a sliding window for local calculation of the speckle contrasts and formation of a contrast map per raw speckle frame. (5) Converting contrast maps to perfusion maps based on a pre-saved lookup table. (6) Formation of a denoised perfusion map by temporal averaging of the whole stacks of perfusion maps. (7) Making a background corrected perfusion map with normalization of the input perfusion map by the background perfusion value.

Acquisition and processing codes in the form of user-interfaces were programmed in MATLAB R2019b (Mathworks)^25^ . The figures were made in MATLAB R2019b (Mathworks)^25^ and grouped in Adobe Illustrator 2018. The seven-step perfusion imaging procedure is summarized in Fig. 2. All the steps are explained in detail in the rest of this section.

#### Step 1: Marker segmentation

**Figure 3:**
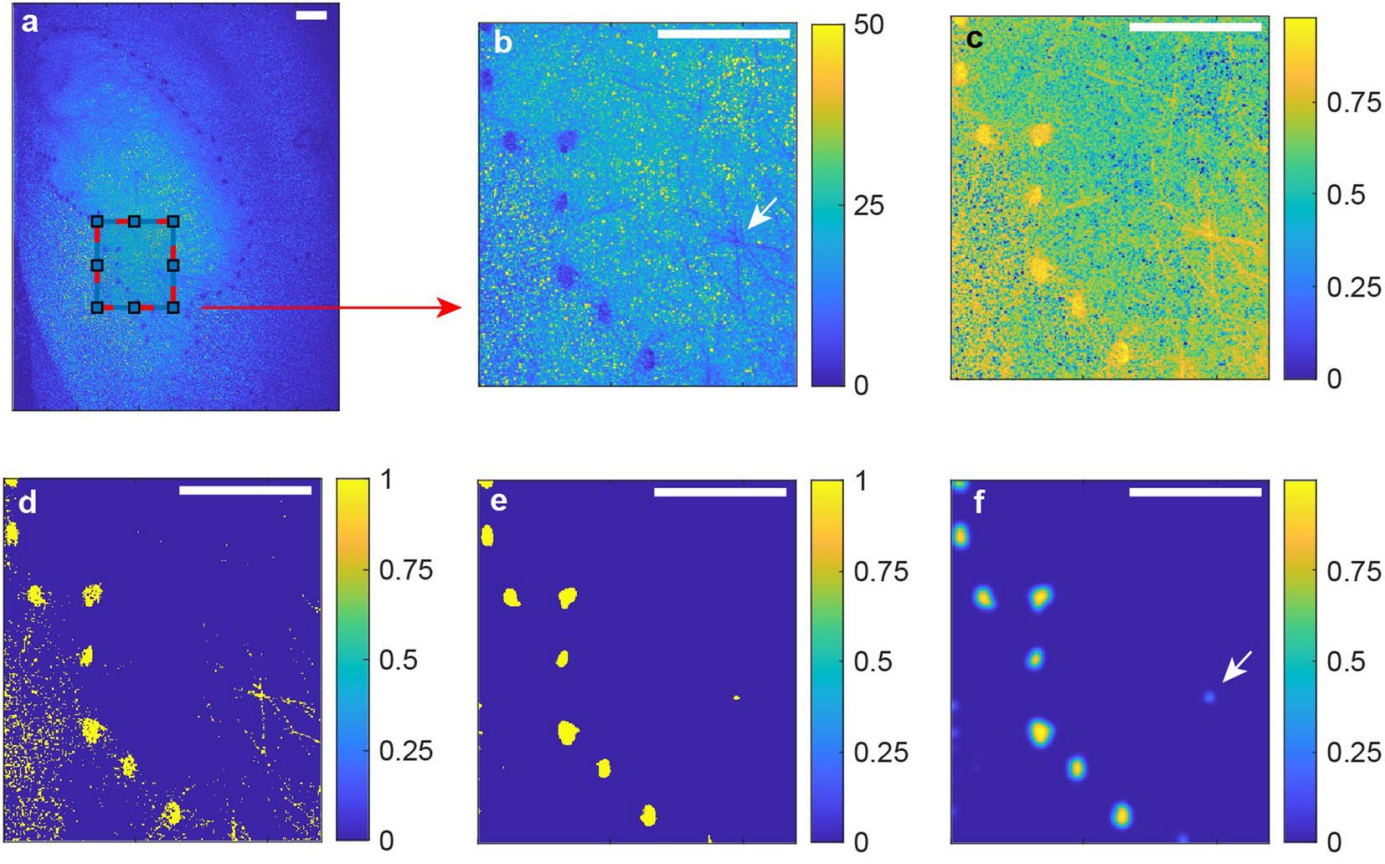
Marker segmentation in a speckle intensity frame. (**a**) Full frame speckle pattern including boundary markers. Scale bars, 10 mm. (**b**) Cropped area shown in (**a**) with a maximum value of 255. (**c**) Normalized and inverted version of (**b**). (**d**) Threshold version of (**c**) including a speckle pattern. (**e**) Mean separation segmentation. (**f**) Gaussian filtering. White arrows indicate a natural landmark formed by intersection of hairs.

A stack of raw speckle frames of a representative handheld measurement including movements of the handheld LSCI system and the patient is shown in Supplementary Video S1. The frames were segmented with the aim to prepare them for tracking and alignment. A region of interest (ROI) was manually selected in the first frame that included boundary-indicating marked points, which was only used for the purpose of marker segmentation and frame alignment (see Fig. 3a,b). Then, a normalized and inverted version of the cropped frame was made with a cut-off value of 25 out of 255 such that the markers had a higher intensity than the surrounding tissue (Fig. 3c). This image had a threshold at 0.8 to provide the segmentation (Fig. 3d). The background speckle pattern differs for each frame and therefore should be removed from all frames. To do so, we applied the MATLAB R2019b (Mathworks)^25^ software package ‘localized active contour’^26,27^, which implements image segmentation in mean separation (MS) mode^28,29^ shown in Fig. 3e with the parameters listed in Table 1.

A Gaussian sliding window of standard deviation 3 was applied to the segmented image (Fig. 3f). Finally, the product of the cropped version shown in Fig. 3b with the Gaussian filtered segmented version shown in Fig. 3f was used for tracking and alignment.

**Table 1:** Parameter setting for the localized active contour segmentation employed in mean separation (MS) mode.

Symbol	Parameter	Value
*N*	Number of iterations	20
*rad*	Side length of the square window	1
$\alpha$	Coefficient to balance the image fidelity and regularization terms	0.09
$\epsilon$	Value for Delta and Heaviside step functions	1

#### Steps 2–3: Marker tracking and frame alignment

After marker segmentation of all frames, the first segmented frame of each experiment was used as the reference frame. Displacements of the rest of the frames with respect to the reference frame were detected by IAT MATLAB R2019b (Mathworks)^25^ toolbox^30^ that works based on maximization of enhanced correlation coefficients^31^. Here, the inputs are: the number of iterations $N=20$; the number of levels for multi-resolution execution 2; the type of geometric transformation ‘translation’; and the initial transformation per frame is the translation elements of the previous frame. This way, a concurrent localization is obtained. The output is a matrix called ‘warp’ that contains horizontal and vertical displacement elements (see Supplementary Video S2). The warp matrix was then used for two purposes; (1) computation of on-surface speeds $(v_x,v_y)$ by time derivation of the horizontal and vertical position elements (*x*, *y*) and (2) full frame alignment of the speckle raw frames by translating each frame based on the corresponding warp matrix elements in the opposite direction (see Supplementary Video S3).

The root-mean-square-error (RMSE) distance for each measurement is calculated as

1 $$\begin{aligned} d_{\mathrm{RMSE}}=\sqrt{\frac{1}{T_m}\int _{0}^{T_m}(x(t)-{\bar{x}})^2+(y(t)-{\bar{y}})^2dt}, \end{aligned}$$

where $T_m$ is the measurement time (approximately 7 s) and $({\bar{x}}, {\bar{y}})$ is the mean location for each experiment. The average on-surface speed per measurement is calculated as

2 $$\begin{aligned} {\bar{v}}=\frac{1}{T_m}\int _{0}^{T_m}{\sqrt{v_x^2(t)+v_y^2(t)}dt}. \end{aligned}$$

For the mounted experiments, since the movements were rather small, the tracking could be done for the whole stacks of raw speckle frames. However, due to the sudden displacements of the probe during some handheld experiments, the tracking algorithm became unstable and therefore only part of those experiments could be aligned and considered for motion and perfusion analysis. A summary of the percentages of aligned frames can be found in Supplementary Tables S1–11.

#### Step 4: Speckle contrast and sliding window

The expression for speckle contrast is^32^

3 $$\begin{aligned} C=\frac{\sigma _I}{{\bar{I}}}, \end{aligned}$$

where $\sigma _I$ and ${\bar{I}}$ are the standard deviation and mean values of intensity fluctuations respectively, observed by a camera. To convert the speckle raw frames into the so-called contrast maps, a window spatially sweeps over every speckle frame individually in order to compute the local contrast values according to Eq. (3). In order to implement the algorithm with an efficient processing time, we have used the sliding convolution technique^33^ with a window size of $9~{\mathrm{px}}\times 9~{\mathrm{px}}~(0.8~{\mathrm{mm}}\times 0.8~{\mathrm{mm}})$. Supplementary Video S4 illustrates the stack of contrast maps calculated after alignment of raw speckle frames. Based on the definition of fully dynamic speckle patterns^32^ and time integrated dynamic speckles only the positive contrast values below unity are valid for consideration. For this reason, the values exceeding unity in speckle contrast are clipped. These are mainly the regions outside the imaged skin area where little light is received by the camera sensor. This results in low mean intensity and therefore the contrast raises above 1 [see Eq. (3)].

#### Step 5: Perfusion estimation model

With random motion of the scatterers and a negative exponential approximation for the auto-correlation function of a field, time integrated dynamic speckle patterns have the contrast^11^

4 $$\begin{aligned} C=\sqrt{\frac{\tau _c}{2T}(1-e^{-\frac{2T}{\tau _c}})}, \end{aligned}$$

where $\tau _c$ and *T* represent correlation and camera integration times, respectively. The correlation time is defined as the time it takes for speckle field auto-correlation to reach 1/*e* of its maximum value. Here, the line-of-sight velocity distribution is approximated to be Lorentzian (i.e. a function exponentially decaying with time and reaches 1/*e* at time $\tau _c$) and single scattering of the detected light is taken into account. The simplest characteristic velocity is defined as^34^

5 $$\begin{aligned} v_c=\frac{\lambda }{2\pi \tau _c}, \end{aligned}$$

where $\lambda$ is the wavelength of light. By definition, $v_c$ is of dimension distance per time. Note that the focus of this work is not to quantify the blood flow or volumetric flux but to study the relative changes of $v_c$ as a function of the observed speckle contrast.

In order to estimate a perfusion value ($P_{\mathrm{est.}}$) by measuring a speckle contrast ($C_{\mathrm{meas.}}$), a lookup table was made as following. An array $v_c$ was formed on the interval $[0, 10^{-3}]~({\mathrm{a.u.}})$. Then, for each element of the array $v_c$ a speckle contrast (*C*) was calculated based on Eqs. (4, 5) with $\lambda =671~{\mathrm{nm}}$ and $T=10~{\mathrm{ms}}$. Since a typical contrast value measured on the calibration suspension is $C_{\mathrm{ref.}}=0.2$, the array $v_c$ was scaled to show a reference (and arbitrary) perfusion $p_{\mathrm{ref.}}=250$ at $C_{\mathrm{ref.}}$. Therefore, the calibrated lookup table was a plot of *C* versus $P=\frac{v_c(T, C_{\mathrm{ref.}})}{p_{\mathrm{ref.}}}v_c\times 10^6$ (see Fig. 5b). The quantity *P* is referred to as perfusion (a.u.) throughout this work. Using this lookup table, stacks of speckle contrast maps were converted to perfusion maps by pixel-based linear interpolation. The reason of creating a lookup table is that $v_c$ cannot be written as a closed-form function of *C*.

**Figure 4:**
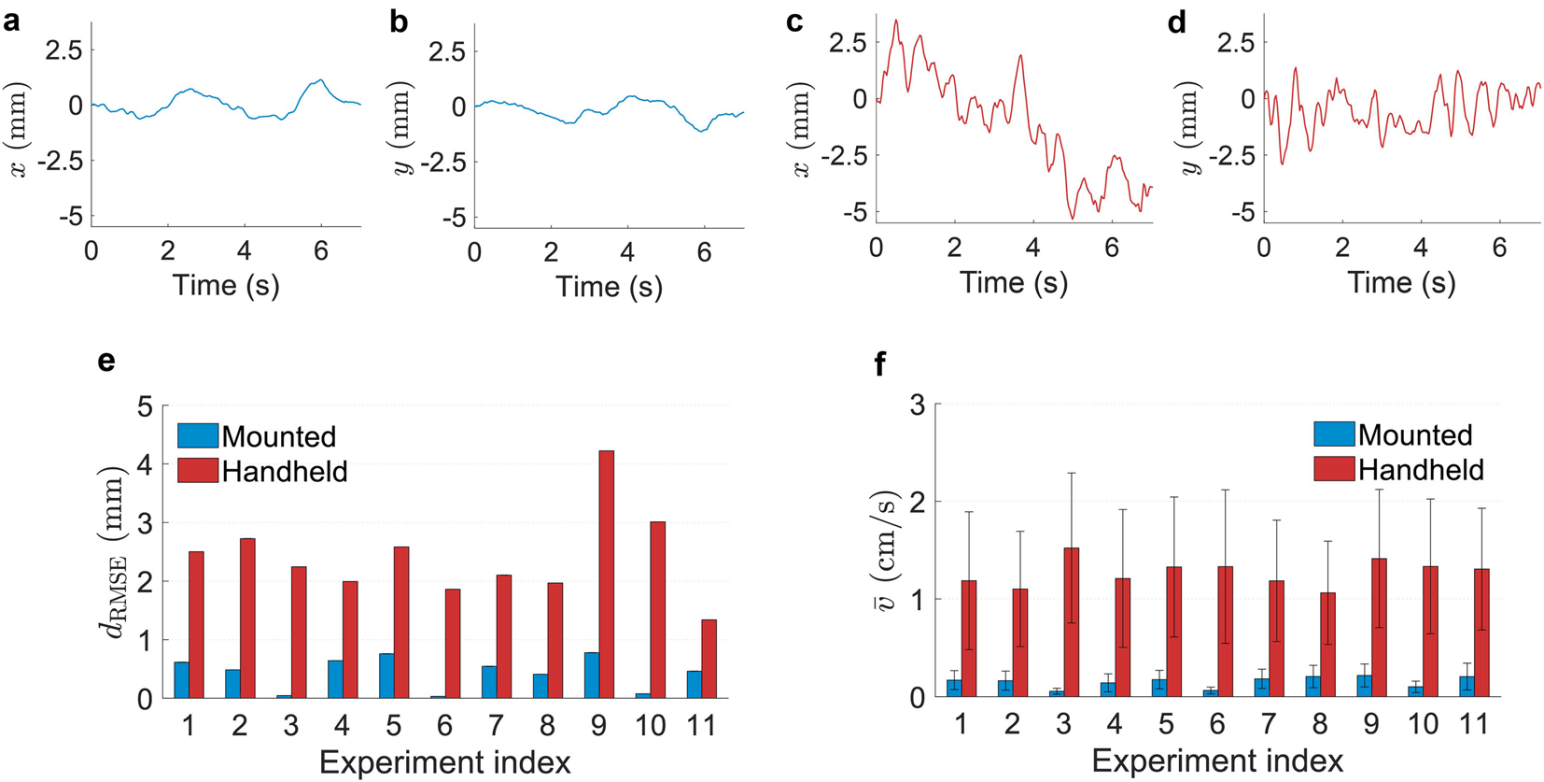
Motion detection with ECC technique and statistical comparison of calculated on-surface speeds for mounted and handheld measurements. Positions of the beam along (**a**) horizontal and (**b**) vertical axes in a mounted experiment. Positive direction in horizontal displacements (*x*) corresponds to right-side orientation in real world coordination. Positive direction in vertical displacements (*y*) corresponds to downside orientation in real world coordination. Positions of the beam along (**c**) horizontal and (**d**) vertical axes in a handheld experiment. In (**a**–**d**) data of experiment index 1 is shown. Overview of the (**e**) root-mean-square-error distance from average location of each experiment and (**f**) on-surface speeds (mean±standard deviation) for the entire time trace of each experiment.

**Figure 5:**
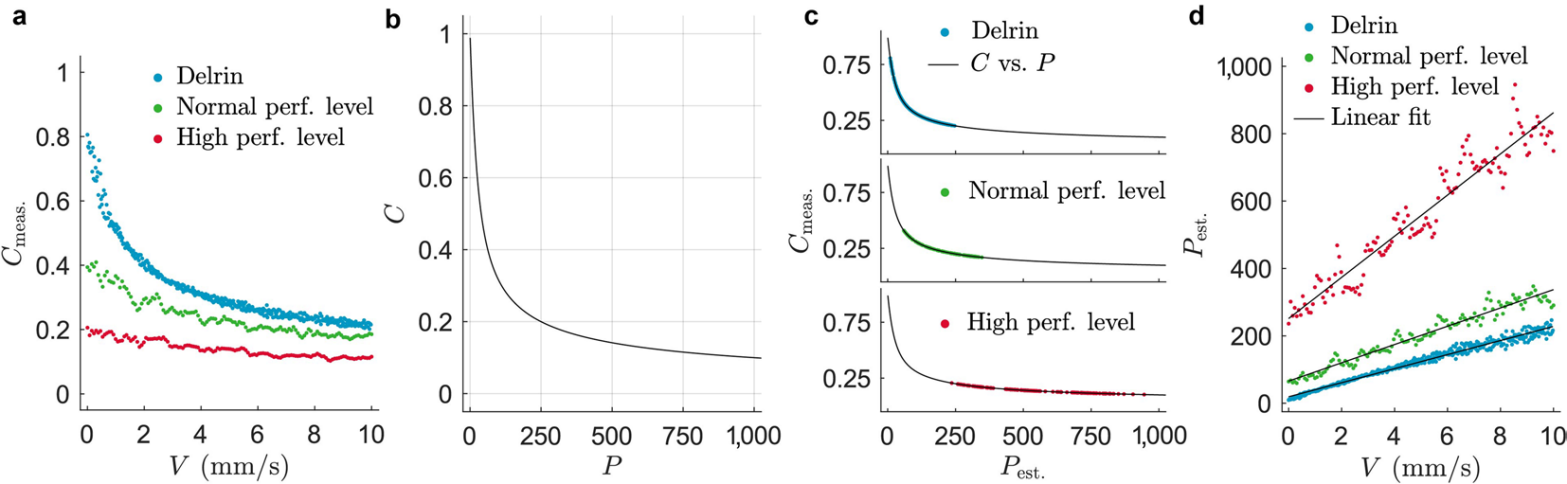
Model examination with motorized displacements of the LSCI system for phantom and in-vivo subjects. (**a**) Measured speckle contrast versus the applied speed. perf.: perfusion. (**b**) Contrast-perfusion lookup table. (**c**) Linear interpolation of the estimated perfusion data points from the measured speckle contrast according to the contrast-perfusion lookup table. vs.: versus. (**d**) Estimated perfusion as a function of the applied speed overlapped with a linear fit for each experiment.

#### Step 6: Temporal averaging

The perfusion maps for each experiment were temporally averaged to make a smoothed perfusion map of reduced speckle noise^35^. Since the temporal resolution is not of interest in this work, averaging of successive frames is a straightforward method of making a representative perfusion map, also with lower influence of heart pulsatility.

#### Step 7: Background correction

As a measure of the ratio between perfusion in skin lesions to that of unaffected skin (background perfusion), a background correction step has been introduced. To do so, three ROIs on the healthy skin around a lesion are manually selected and the average perfusion of the selected areas, i.e. background perfusion $\bar{p_b}$, is calculated. Then, the whole temporally averaged perfusion map is divided by $\bar{p_b}$ in order to form the background corrected perfusion map^7^ where the background perfusion is approximately 1 (Supplementary Tables S1–11).

### Processing times

The processing times can be categorized in measurement time and post processing time. The measurements were carried out with a processor Intel(R) Core(TM) i7-3770 CPU @ 3.4 GHz and RAM capacity of 16 GB. The total time for a single measurement and saving was $49\pm 1$ s. The program shows perfusion maps (with a sliding window size of $21~{\mathrm{px}}\times 21$ px on a frames size of $1536~{\mathrm{px}}\times 2048$ px) in a preview mode with a rate of $1.46\pm 0.02$ Hz.

The post-processing of the measurements was carried out on a desktop computer with a processor Intel(R) Core(TM) i7-8700K CPU @ 3.70 GHz with a RAM capacity of 16 GB. A sample segmentation and alignment in a region of $175~{\mathrm{px}}\times 240$ px took 129 s. The temporal averaging of all 211 frames of a measurement took 109 s. The background correction took 45 s. Note that the post processing time not only depends on the computer performance, but also it depends on the user skills in loading files and choosing regions in an image. Therefore, the entire post-processing of a measurement can be performed in 5 min.

### Statistical analysis

The perfusion of handheld and mounted measurements was analyzed using descriptive statistics. In addition, paired t-tests were performed between all temporally averaged mounted-handheld measurement pairs. This was done for the mean lesion perfusion in measurements pairs both before (temporally averaged values) and after background correction. Also, the intraclass correlation coefficients (ICCs), a measure for the amount of agreement with a value of 1 reflecting perfect agreement, were calculated for the aforementioned measurement pairs. Statistical analyses were performed with Statistical package SPSS, version 25 (IBM). A two-sided $p<0.05$ was considered statistically significant.

### In-vitro and in-vivo evaluation of the perfusion estimation model in a healthy volunteer

For the in-vitro experiment, the handheld LSCI system was mounted on a translational stage facing perpendicular to a Delrin plate and a linear speed range of $V=[0,10]$ mm/s during 10.35 s was applied. An example of this experiment is shown in Supplementary Video S5 for an exposure time of $T=10\,{\mathrm{ms}}$.

Similarly, for the in-vivo experiments, that were performed before usage in psoriasis patients, the LSCI system was mounted on a translational stage to which a linear speed range of $V=[0,10]$ mm/s during 3.3 s was applied. The study consisted of two phases. In the first phase, the skin had a normal perfusion level. In the second phase, the measurement was carried out 15 min after applying 0.2 ml vasodilating cream (60 gr Midalgan cream Extra Warm, Qualiphar, Meppel, The Netherlands) on an area of $20~{\mathrm{cm}}\times 5~{\mathrm{cm}}$. The second phase is referred to as ‘high perfusion level’.

For both the in-vitro and in-vivo experiments, a $20^\circ$ top hat engineered diffuser (Thorlabs ED1-S20-MD) with square scattered shape was used to expand the laser beam. The speckle frames were captured with an exposure time of $T=25\,{\mathrm{ms}}$, a frame rate of 40 Hz and a gain of 0 dB. The speckle contrast for each captured frame was calculated from a windows of size $150~{\mathrm{px}}\times 150$ px.

## Results

### Magnitude of movements

Relative displacements of the camera and targeted markers around the lesions were observed in all experiments. In mounted experiments displacements are caused by patient movements and in handheld experiments displacements are caused by a combination of the probe and the patients movements. Localization and on-surface speed calculation for the handheld measurement of experiment index 1 has been demonstrated in Supplementary Video S2. Figure 4a,b illustrates horizontal and vertical positions with respect to the first frame for a representative mounted experiment. The RMSE-distance and the average on-surface speed values for this experiment are 0.6 mm and 1.7 mm/s, respectively. The corresponding handheld experiment has been depicted in Fig. 4c,d with an RMSE-distance and average on-surface speed values of 2.5 mm and 11.9 mm/s, respectively.

Figure 4e gives an overview of comparison between the RMSE-distance with an average of 0.4 mm and 2.4 mm for all of the mounted and handheld measurements, respectively. In Fig. 4f, the on-surface speeds are depicted which are calculated based on Eq. 2. The averages of the absolute on-surface speed values (Eq. 2) shown in Fig. 4f are 1.5 mm/s and 12.7 mm/s for the mounted and handheld measurement pairs, respectively, which shows an increase with a factor of 8.5.

### Contrast-perfusion model examination

The purpose of the model examination is to explore the estimated perfusion response to a linear increase of the applied speed using the contrast-perfusion lookup table described in Methods *Step 5: Perfusion estimation model* . Figure 5a illustrates the measured speckle contrast versus the applied speed observed on a static scattering medium (Delrin) and on a forearm of a healthy subject with both normal and high perfusion levels. The following can be observed. (1) The speckle contrast at zero-speed is not unity for Delrin as expected theoretically since Delrin is a static object; (2) The measured speckle contrast decays non-linearly as a function of the applied speed for all three cases; (3) For the same applied speed profile, the dynamic range of the speckle contrast decreases as the subject includes more internal movements.

**Figure 6:**
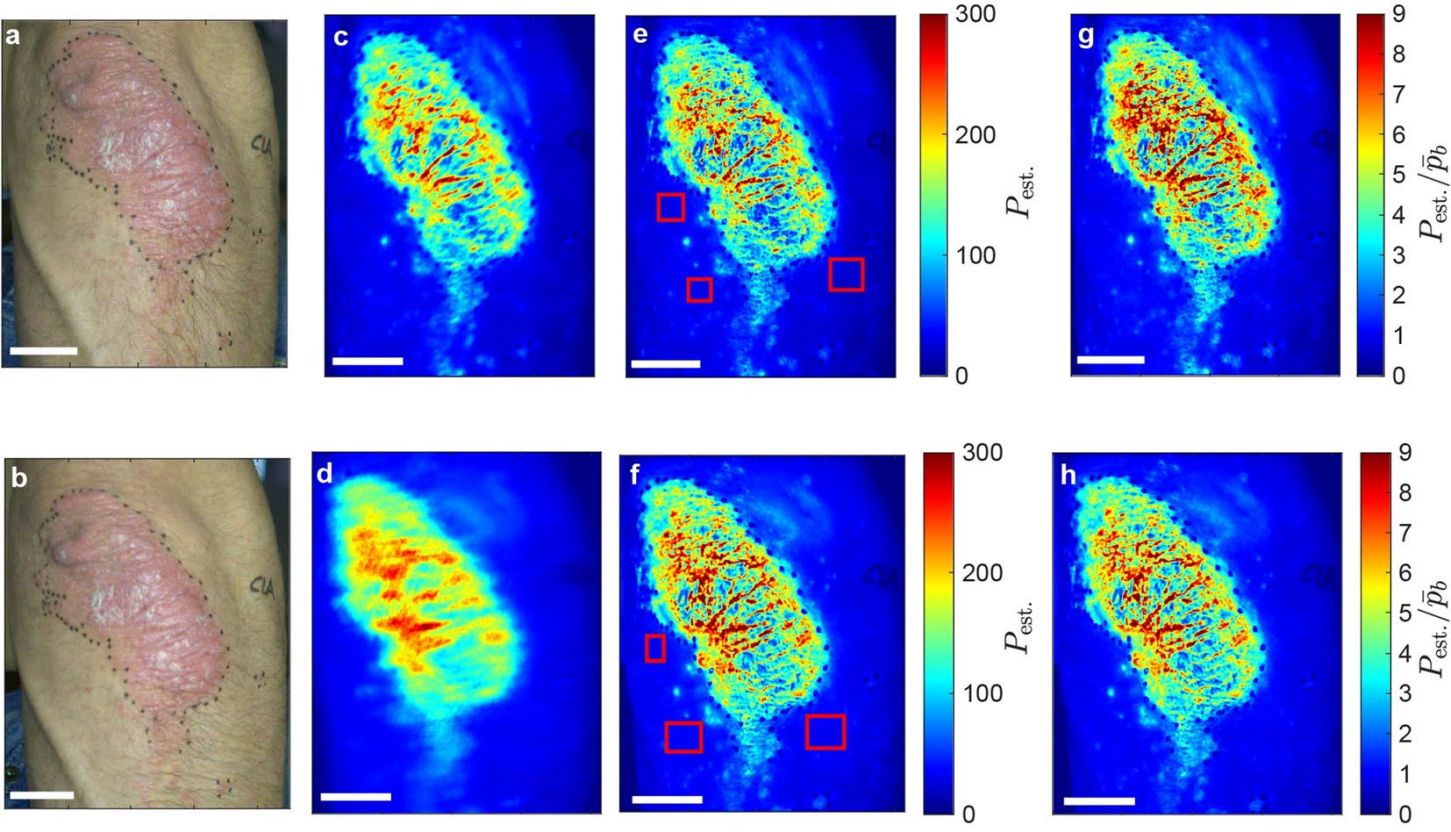
Visual comparison of a representative mounted (top row) and the corresponding handheld (bottom row) experiments. (**a**,**b**) RGB photographs. Scale bars, 25 mm. Temporally averaged perfusion maps (**c**,**d**) before and (**e**,**f**) after the alignment. Red rectangles indicate areas in which background perfusion values (${\bar{p}}_b$) are calculated. $P_{\mathrm{est.}}$: estimated perfusion. (**g**,**h**) Background corrected perfusion maps. Data of experiment index 1 is shown.

The predefined contrast-perfusion curve is shown in Fig. 5b which was introduced in Eqs. (4, 5). Using this curve a perfusion value is assigned to each measured speckle contrast data point. The set of estimated perfusion data points for the three experiments is depicted in Fig. 5c that gives us a visual impression to which part of the curve these data points belong. Finally, a plot of estimated perfusion versus the applied speed is shown in Fig. 5d where a rather linear relation for each case is observed. This relation is investigated by a fit function as;

6 $$\begin{aligned} P_{\mathrm{est.}}=aV+b. \end{aligned}$$

The pair of (*a*, *b*) for Delrin, low and high perfusion levels are obtained as (20.9, 19.1), (27.1, 65.7) and (61.1, 251.4), respectively. The $R^2$ values for Delrin, low and high perfusion levels are 0.98, 0.95 and 0.92, respectively. The *y*-intercept (*b* value) of this fit function points at two issues: (1) experimental imperfection (i.e. the fact that *b* is non-zero for Delrin) which can be due to small vibration of the setup, camera noise, and laser source; and (2) baseline perfusion (i.e. perfusion level without movement artefact). Also, both the slopes and *y*-intercepts increase as the internal movements of the subjects increase. Note that the fluctuations in high perfusion level shown in Fig. 5d are noticeably driven by the heart pulsations.

### RGB imaging and visual comparison of perfusion maps

The RGB image of a mounted measurement is shown in Fig. 6a where the clinically visible lesion and boundary-indicating black marking points are apparent. The temporally averaged perfusion maps of this measurement before and after the alignment are illustrated in Fig. 6c,e, respectively. Although there is no movement of the handheld LSCI system during this mounted measurement, patient movement causes slight blurring of this perfusion map before the alignment. The healthy skin around the psoriasis lesion has a lower perfusion in comparison to the lesion. The low intensities of the black markers make the speckle contrasts exceed unity; thus, the corresponding perfusion values become close to zero. The associated background corrected perfusion map is depicted in Fig. 6g with a background perfusion level of approximately 1. The corresponding RGB image, temporally averaged and background corrected perfusion maps for the handheld experiment are demonstrated in Fig. 6b,d,f,h, respectively. For an overview of the entire collection of measurement pairs, see Supplementary Figs. S1–11.

### Influence of alignment on spatiotemporal perfusion profiles

**Figure 7:**
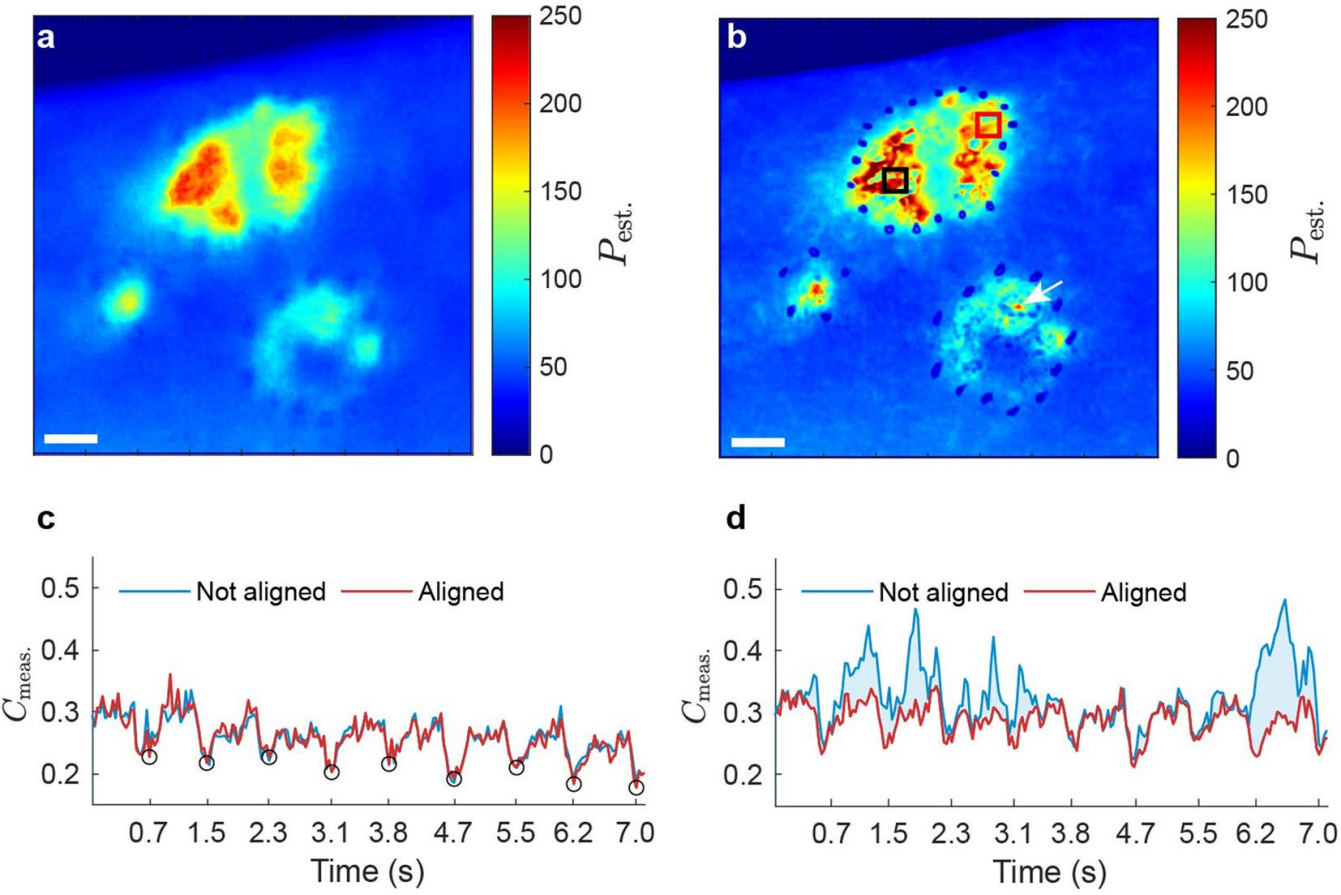
Spatiotemporal analysis of speckle frames alignment. Data of experiment index 6 is shown. (**a**) Temporally averaged perfusion map of a representative handheld measurement without alignment. $P_{\mathrm{est.}}$: estimated perfusion. Scale bars, 10 mm. (**b**) Temporally averaged perfusion map of the same measurement after alignment. White arrow indicates a small region with a rather higher perfusion value which is not distinguishable without alignment. (**c**) Temporal fluctuation of the measured speckle contrast ($C_{\mathrm{meas.}}$) during the corresponding handheld measurement with and without alignment. The region of interest for the calculation is shown as the black square in (**b**). Open circles: manually selected sudden drops in the speckle contrasts possibly driven by the heartbeats. (**d**) Measured speckle contrast versus time during the handheld measurement where the calculation region is shown as the red square in (**b**).

A temporally averaged handheld perfusion map without alignment is shown in Fig. 7a. This perfusion map is blurred due to the movements. The aligned perfusion map is shown in Fig. 7b, in which the marking points look sharper. Two regions are selected to examine the temporal fluctuation of the speckle contrast. The first ROI (the black square shown in Fig. 7b) is placed far from the lesion boundaries. The contrast of this region in the time domain with and without alignment is shown in Fig. 7c, where the graphs are to some extent similar with a root mean square error (RMSE) of 0.02. The dips in the speckle contrast pattern are a suggestive heartbeat signal with an estimated rate of 77 beats/min. A second ROI was selected in a region close to the lesion boundary (the red square shown in Fig. 7b) and the speckle contrast time traces were computed (see Fig. 7d). For a demonstration of the analysis without and with tracking, see Supplementary Video S6 and Supplementary Video S7, respectively. This time, at the interval when the ROI overlaps the markers or goes beyond the lesion area, the calculation of speckle contrast is affected and could be unreliable when the region is not tracked. The RMSE for this pair is 0.06 which is, in this example, 3 times of that on the first ROI. It is worth noting that the suggestive heartbeat pattern could not be detected in all measurements even in mounted mode.

**Figure 8:**
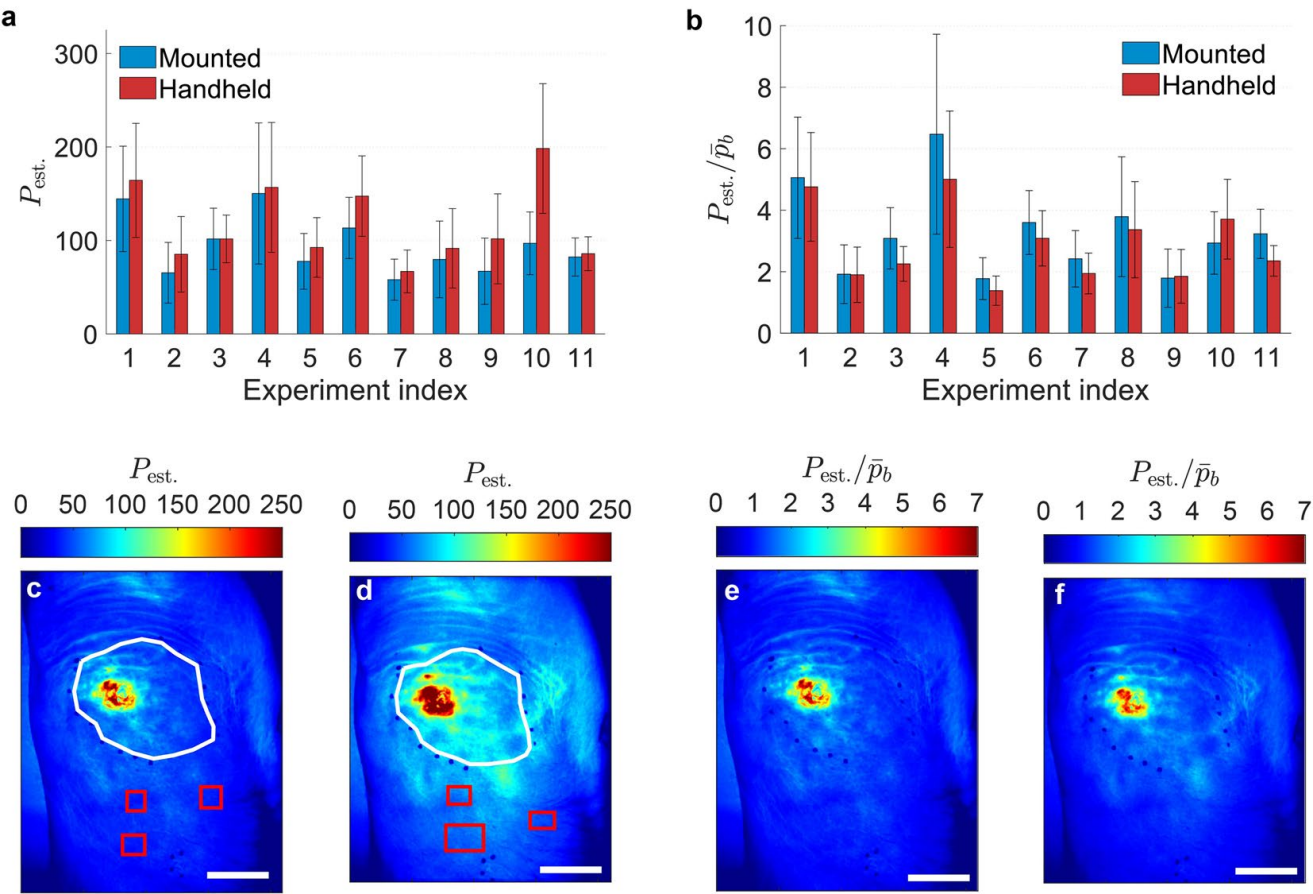
Visualization and statistical comparison between temporally averaged and background corrected perfusion maps per pairs of mounted and handheld experiments. Overview of average perfusion over the entire lesion for (**a**) temporally averaged and (**b**) background corrected perfusion maps. $P_{\mathrm{est.}}$: estimated perfusion; ${\bar{P}}_b$: background perfusion. Data are reported as mean±standard deviation. Representative temporally averaged perfusion maps of (**c**) mounted and (**d**) handheld measurements of experiment index 9. Red rectangles: manually selected regions in which background perfusion values (${\bar{P}}_b$) are calculated. White polygons: manually selected lesion areas. Background corrected perfusion maps of corresponding (**e**) mounted and (**f**) handheld measurements. Scale bars, 25 mm.

### Accounting for movement artefacts and effect of background correction

A comparison between temporally averaged and background corrected perfusion maps gives a measure of similarity between the pairs of mounted and handheld measurements. Fig. 8a,b summarizes the mean perfusion values of the entire lesion indicated by black marking points on the skin for all measurement pairs (Supplementary Figs. S1–11 ). It is worth noting that the collections $\{1,4,8\}$, $\{3,6,10\}$, $\{5,9\}$, $\{7,11\}$ and $\{2\}$ belong to same patients captured in different days, i.e. data of 5 patients are illustrated.

In all experiments, the temporally averaged handheld measurements (Fig. 8a) showed greater mean perfusions. The temporally averaged perfusions for mounted and handheld measurements are $94.2\pm 29.5$ (mean±std, $n=11$) and $117.4\pm 40$ (mean±std, $n=11$) with medians of 82.3 and 101.6, respectively. An absolute difference of $26.2\pm 28.5~\%$ (mean±std, $n=11$), and an absolute median difference of $23.4\%$ exist between the mounted and handheld temporally averaged perfusion map pairs due to the movement artefacts. A paired t-test revealed that the difference between the handheld and mounted pairs of temporally averaged perfusion maps is statistically significant ($p=0.02$).

The mean perfusions of the corresponding lesions in the background corrected perfusion maps are shown in Fig. 8b. The regions chosen for background correction are provided in Supplementary Figs. S1–11 . The background corrected perfusions for mounted and handheld cases are $3.3\pm 1.4$ (mean ± std, $n=11$) and $2.9\pm 1.2$ (mean ± std, $n=11$) with medians of 3.1 and 2.3, respectively. After the background correction, the absolute difference between the mounted and handheld pairs becomes $16.4\pm 9.3~\%$ (mean ± std, $n=11$), with an absolute median difference of $23.8\%$. A paired t-test revealed that the difference between the handheld and mounted measurement pairs after the background correction remains statistically significant ($p=0.04$).

Therefore, background correction helps to decrease the absolute difference between the mounted and handheld pairs caused by movement artefacts on average from 26.2 to $16.4\%$ although such improvement is negligible when median values are considered. These movement-induced difference margins are to be considered when analyzing handheld perfusion maps. The paired t-tests revealed that background correction does not guaranty that the mounted-handheld pairs become insignificantly different. However, the ICCs for the aforementioned pairs were 0.603 (95% CI 0.02–0.877, corresponding with moderate agreement) and 0.874 (95% CI 0.514–0.967, corresponding with good agreement) for the temporally averaged and background corrected pairs, respectively. This suggests that background correction increases the agreement between the mounted-handheld measurement pairs.

In Fig. 8c–f a visual example is shown in which, having the same color scale, the pair of background corrected images are more comparable to each other. Note that the average perfusion values of the handheld measurements after the background correction shown in Fig. 8b are, mostly, lower than the mounted measurements. To elaborate on the reason, we argue the following. (1) If in the entire image, the perfusion value is increased with a constant value, the relative increase for the background perfusion (which is low) exceeds the relative increase of the lesion value (which is high). (2) This effect is mitigated since the motion-induced perfusion increment increases with the perfusion (as shown in Fig. 5d, however this effect cannot fully undo the effect of (1)).

## Discussion

The performance of handheld LSCI was studied in detail and the outputs were compared with mounted experiments in psoriasis patients using the HAPI prototype (Fig. 1). We introduced a post-processing procedure of handheld LSCI for step-by-step analysis of acquired raw speckle frames to reach a representative perfusion map which matches the mounted perfusion map (Fig. 2). The advantage of frame alignment in LSCI was previously shown by Richards et al.^36^ intraoperatively and in a mounted modality. In this study, we employed segmentation and image alignment for LSCI on psoriasis lesions in a handheld modality. The size of a selected ROI for aligning handheld measurement frames should not be too large as an account for the computation time, and not too small, as an account for having stable results.

For tracking and image alignment, the geometric transformation is chosen as ‘translation’ which means that the rotations are ignored. As a consequence, the aligned sequence of speckle frames include residual movements in the form of rotations which is apparent in Supplementary Video S3 although the temporally averaged perfusion maps are sharp enough to neglect the rotations. Despite the ability of the employed ECC image alignment method to account for rotational transformation as well as translational, the rotational transformation cannot be done since the center point for an ROI is not necessarily the same as the center point of the whole frame. Although several state-of-the-art methods for segmentation and alignment exist, the combination of MS segmentation and ECC image alignment for this work has been chosen since they are robust, computationally cheap and can effectively remove the speckle patterns in the background. Here a proof-of-concept for alignment and motion detection within the speckle intensity frames is shown, that highlights the potential of this method for other handheld applications or those including patient movements in the field.

The existence of (natural or artificial) markers in the imaged field is a key requirement for optimal perfusion imaging and perfusion image alignment. In this study, black marking points were applied on the skin. Naturally, for the applicability in daily clinical practice, the use of natural (skin) landmarks would be preferable. Figure 3f shows an example of the detection of a crossing hair as a natural landmark. However, the possibility and accuracy of tracking natural landmarks with the current algorithm remains a challenge. Alternative solutions such as employing optical-flow algorithm to track the natural landmarks on RGB frames acquired parallel to the speckle frames might be of help.

Relative movements between the handheld probe and subjects are quantified based on the RMSE-distance analysis (Fig. 4e). The RMSE-distance is a measure of displacements of the acquired frames from the average point (center of gravity). Since the RMSE-distances for both handheld and mounted modes are non-zero, the frame alignment is a required step in order to prevent blurring of the temporally averaged perfusion maps as well as obtaining a reliable temporal perfusion profile when choosing an area on a perfusion map. The average absolute on-surface speed for handheld measurements is calculated as 8.5 times greater than the mounted ones (Fig. 4f). In a previous study, the average on-surface speed for handheld measurements by 10 healthy operators on a static object was calculated as 9 mm/s where the measurement distance was 20 cm^18^. In this study, the calculated value is 12.7 mm/s with the measurement distance of 40 cm. The increase in the calculated value in this study is due to the added movements of the patients during measurements as well as the increased measurement distance.

The exact relationship between speckle contrast and blood flow is open for research and has been addressed in a range of articles^37,38^. The theoretical model that we have used to estimate the perfusion assumes a Lorentzian velocity distribution. Although a modified version of this model is proposed by Duncan et al.^39^, we used it in its native version [Eqs. (4, 5)] and examined it via in-vitro and in-vivo experiments. We observed a linear relation of the estimated perfusion versus the applied speed and the slope of these lines are not necessarily the same for low and high perfusion levels (see Fig. 5d). These results cannot be generalized to any human subject due to the complexity of the human body and change of perfusion from one subject to another. However, what these results suggest is that the movement-related perfusion can be subtracted from the measured perfusion in both low and high levels provided that a range of on-surface speeds is accurately measured. This method would make the procedure of movement artefact correction independent of attaching an opaque surface on the tissue for measuring the speckle contrast driven by involuntary movements.

Three ways for presentation of mounted and handheld experiments have been introduced. First, comparing the pairs of temporally averaged perfusion maps with a localized color-map scaling (Supplementary Figs. S1–11 ). Here the best spatial similarity is achieved compared to the other two ways mentioned in the following. Second, comparing the pairs of temporally averaged perfusion maps with the same color-map scaling (Fig. 8c,d and Supplementary Figs. S1–11 ). In this case, the influence of movement-related perfusion is apparent. Depending on the magnitude of movements, handheld measurements are of higher average perfusion values. Third, comparison of the background corrected pairs with the same color-map scaling (Fig. 8e,f and Supplementary Figs. S1–11 ). This step helps to know the perfusion level in a lesion relative to the background perfusion. Moreover, in the analysis of this work, all captured frames were used for temporal averaging. As an improvement, one can choose certain frames with the lowest average perfusion in order to decrease the influence of heartbeat and movement artefact in the temporally averaged perfusion maps. However, based on the type of the analysis and the required accuracy, compensation for movement artefacts may still be necessary for some applications.

While our post-processing procedure is effective in obtaining perfusion maps that are less influenced by involuntary movements, artefacts caused by blurring of the speckle patterns within the exposure time are not corrected. For exact correction of the speckle contrast loss due to motion, both on-surface speed and tilt of wavefronts are required as well as knowledge about optical properties of the tissue. Another factor contributing to speckle blurring within the exposure time is moderately high frequency (i.e. around 40 Hz) and low amplitude (i.e. about 1 px) vibrations that are conducted to the patient’s skin (e.g. vibrations transferred from the floor to the skin). This factor poses potential limitation in obtaining a movement-artefact-free measurement. Also, power spectral analysis (PSD) of time-variant local perfusion helps to identify and correct for the influence of cardiac and respiratory rhythms.

## Conclusion

We have examined handheld LSCI in a clinical research setting by introducing a post-processing procedure and made a comparison with mounted measurements. Our results show that handheld measurements are reliable in terms of visual similarity compared to mounted measurements. Enabling handheld perfusion imaging would be highly beneficial for implementation in daily clinical practice. The compact and portable HAPI probe realizes a comfortable skin perfusion measurement for both patients and medical staff. The background correction reduced the absolute difference between the mounted and handheld pairs to $16.4\pm 9.3\%$ (mean±std, $n=11$), with an absolute median difference of $23.8\%$. Although the differences between the mounted-handheld pairs in both temporally averaged and background corrected cases remain statistically significant, the degree of agreement between them increases after the background correction. Depending on the application in a clinical practice, this difference induced by movement artefacts may be acceptable. The results of this study can be used to advance the LSCI technology towards clinical use, especially for correction of movement artefacts in the handheld mode.

## Supplementary Information

Supplementary Information.

Supplementary Code S1.

Supplementary Video S1

Supplementary Video S2

Supplementary Video S3

Supplementary Video S4

Supplementary Video S5

Supplementary Video S6

Supplementary Video S7

## Data Availability

The datasets generated during and/or analyzed during this study to reproduce the graphs within the manuscript (Figs. 2, 3, 4, 5, 6, 7, 8) are available in the Figshare repository, https://doi.org/10.6084/m9.figshare.14995083. The temporally averaged and background corrected perfusion maps for experiment indices 2-5, 7-8 and 10-11 are available from the corresponding author on reasonable request. The raw speckle frames for experiment indices 2-11 are available from the corresponding author on reasonable request.

## Code availability

The codes used for analysis of the raw data as user-interface during the current study are available in Supplementary Code S1.
