# Supplementary material for: Handheld versus mounted laser speckle contrast perfusion imaging demonstrated in psoriasis lesions
Source: Sci Rep. 2021 Aug 17;11:16646. doi: 10.1038/s41598-021-96218-6 (PMC8371022; doi:10.1038/s41598-021-96218-6)
Supplement: Supplementary file 1 — Supplementary Information. [file 41598_2021_96218_MOESM1_ESM.pdf]

Supplementary information:
Handheld versus mounted laser speckle contrast perfusion imaging demonstrated in psoriasis lesions

Ata Chizari[a,*,+], Mirjam J. Schaap[b,+], Tom Knop[a], Yoeri E. Boink[a,c,d], Marieke M.B. Seyger[b], and Wiendelt Steenbergen[a]

[a]Biomedical Photonic Imaging, Technical Medical Centre, Faculty of Science and Technology, University of Twente, Enschede, The Netherlands, P.O. Box 217, 7500 AE
[b]Department of Dermatology, Radboud University Medical Center, Nijmegen, The Netherlands, P.O. Box 9101, 6500 HB
[c]Multi-Modality Medical Imaging, Technical Medical Centre, Faculty of Science and Technology, University of Twente, Enschede, The Netherlands, P.O. Box 217, 7500 AE
[d]Department of Applied Mathematics, University of Twente, Enschede, The Netherlands, P.O. Box 217, 7500 AE
[*]email: a.chizari@utwente.nl
[+]These authors contributed equally

ABSTRACT

This document includes detail information for the 11 pairs of handheld and mounted experiments, namely measurement time, percentage of aligned frames, average perfusion in the selected background areas, mean intensity on Delrin, speckle contrast on Delrin and speckle contrast on scattering suspension. For each experiment index, the pairs of temporally averaged and background corrected perfusion maps are also illustrated.

Experiment index 1

	Time	% Aligned frames	$\bar{p}_b$	$\bar{I}$	C_s	C_d
Mounted	9 : 34	100	28.6	15.9	0.86	0.17
Handheld	9 : 41	100	34.5			

Supplementary Table S 1. Detail information of the measurement pair. $\bar{p}_b$: Average perfusion in the selected background areas. $\bar{I}$: Mean intensity on Delrin out of 255. C_s: Speckle contrast on Delrin. C_d: Speckle contrast on scattering suspension.

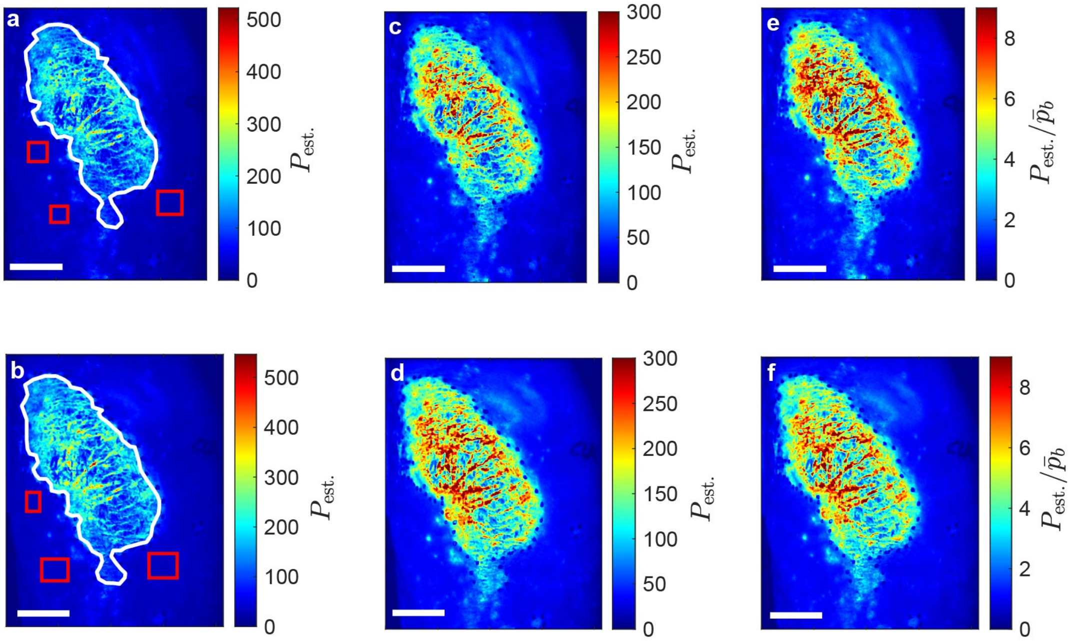

Supplementary Figure S 1. Comparison of mounted (i.e. (a), (c) and (e)) and handheld (i.e. (b), (d) and (f)) measurements. $P_{\mathrm{est.}}$: estimated perfusion. Scale bars, 25 mm. Red rectangles: manually selected regions for calculation of background perfusion ($\bar{p}_b$). White polygons: manually selected lesion areas. Temporally averaged perfusion maps with (a-b) a localized color-map scaling and (c-d) the same color-map scaling. (e-f) Corresponding background corrected perfusion maps with the same color-map scaling.

Experiment index 2

	Time	% Aligned frames	$\bar{p}_b$	$\bar{I}$	C_s	C_d
Mounted	9 : 33	100	34	13.5	0.8	0.16
Handheld	9 : 35	100	44.9			

Supplementary Table S 2. Detail information of the measurement pair. $\bar{p}_b$: Average perfusion in the selected background areas. $\bar{I}$: Mean intensity on Delrin out of 255. C_s: Speckle contrast on Delrin. C_d: Speckle contrast on scattering suspension.

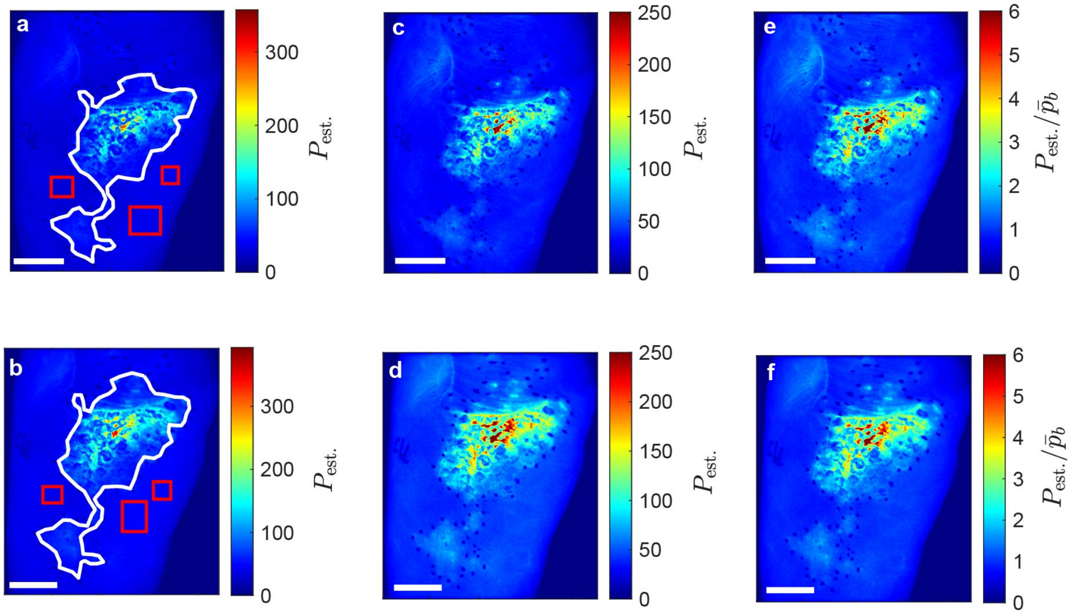

Supplementary Figure S 2. Comparison of mounted (i.e. (a), (c) and (e)) and handheld (i.e. (b), (d) and (f)) measurements. $P_{est.}$: estimated perfusion. Scale bars, 25 mm. Red rectangles: manually selected regions for calculation of background perfusion ($\bar{p}_b$). White polygons: manually selected lesion areas. Temporally averaged perfusion maps with (a-b) a localized color-map scaling and (c-d) the same color-map scaling. (e-f) Corresponding background corrected perfusion maps with the same color-map scaling.

Experiment index 3

	Time	% Aligned frames	$\bar{p}_b$	$\bar{I}$	C_s	C_d
Mounted	13 : 32	100	33	21.9	0.9	0.2
Handheld	13 : 41	66	45.1			

Supplementary Table S 3. Detail information of the measurement pair. $\bar{p}_b$: Average perfusion in the selected background areas. $\bar{I}$: Mean intensity on Delrin out of 255. C_s: Speckle contrast on Delrin. C_d: Speckle contrast on scattering suspension.

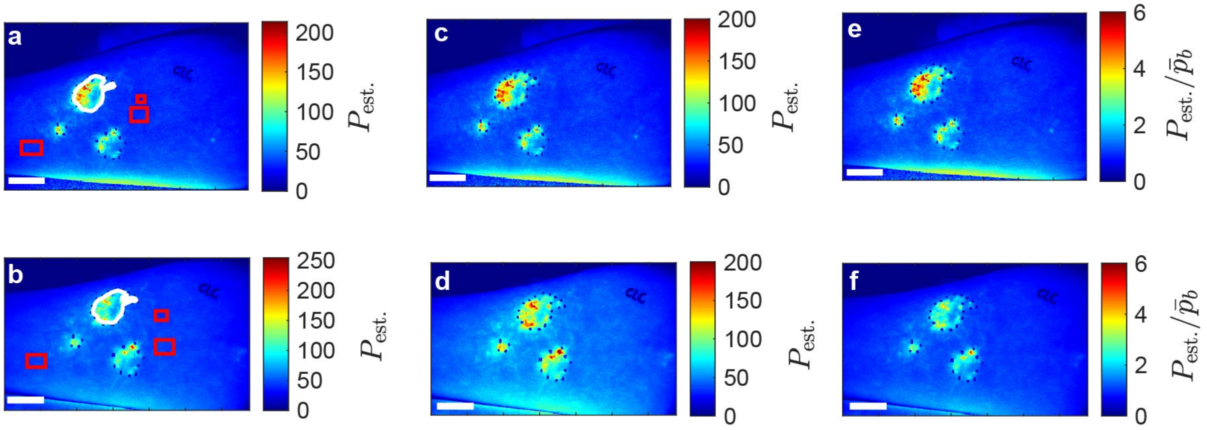

Supplementary Figure S 3. Comparison of mounted (i.e. (a), (c) and (e)) and handheld (i.e. (b), (d) and (f)) measurements. $P_{est.}$: estimated perfusion. Scale bars, 25 mm. Red rectangles: manually selected regions for calculation of background perfusion ($\bar{p}_b$). White polygons: manually selected lesion areas. Temporally averaged perfusion maps with (a-b) a localized color-map scaling and (c-d) the same color-map scaling. (e-f) Corresponding background corrected perfusion maps with the same color-map scaling.

Experiment index 4

	Time	% Aligned frames	$\bar{p}_b$	$\bar{I}$	C_s	C_d
Mounted	9 : 19	100	23.2	19	0.91	0.2
Handheld	9 : 16	100	31.3			

Supplementary Table S 4. Detail information of the measurement pair. $\bar{p}_b$: Average perfusion in the selected background areas. $\bar{I}$: Mean intensity on Delrin out of 255. C_s: Speckle contrast on Delrin. C_d: Speckle contrast on scattering suspension.

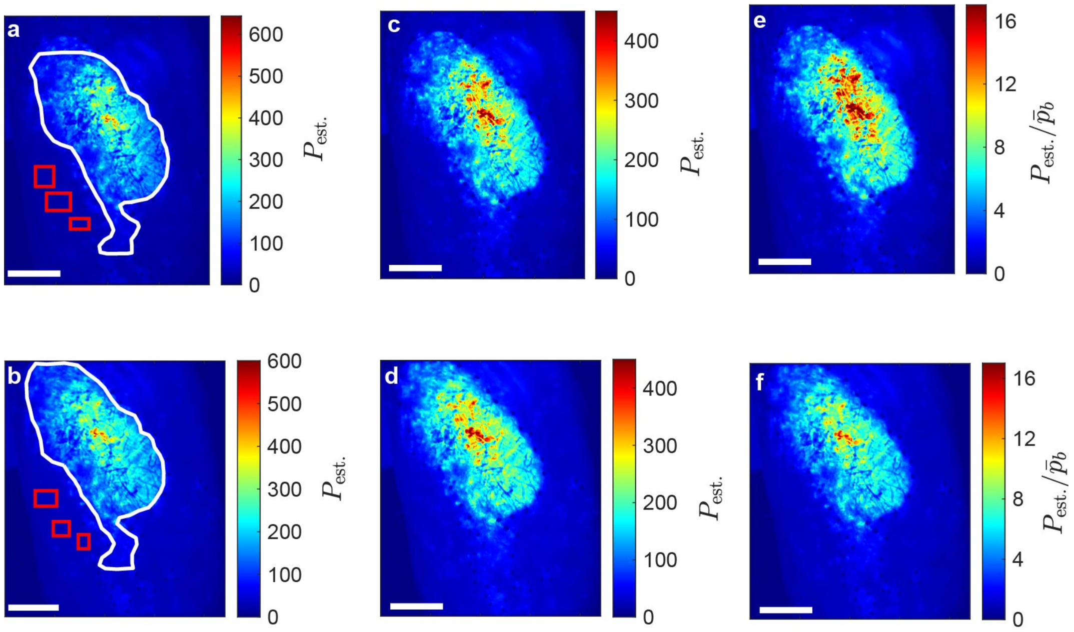

Supplementary Figure S 4. Comparison of mounted (i.e. (a), (c) and (e)) and handheld (i.e. (b), (d) and (f)) measurements. $P_{est.}$: estimated perfusion. Scale bars, 25 mm. Red rectangles: manually selected regions for calculation of background perfusion ($\bar{p}_b$). White polygons: manually selected lesion areas. Temporally averaged perfusion maps with (a-b) a localized color-map scaling and (c-d) the same color-map scaling. (e-f) Corresponding background corrected perfusion maps with the same color-map scaling.

Experiment index 5

	Time	% Aligned frames	$\bar{p}_b$	$\bar{I}$	C_s	C_d
Mounted	13 : 13	100	43.8	16.6	0.89	0.19
Handheld	13 : 11	100	66.9			

Supplementary Table S 5. Detail information of the measurement pair. $\bar{p}_b$: Average perfusion in the selected background areas. $\bar{I}$: Mean intensity on Delrin out of 255. C_s: Speckle contrast on Delrin. C_d: Speckle contrast on scattering suspension.

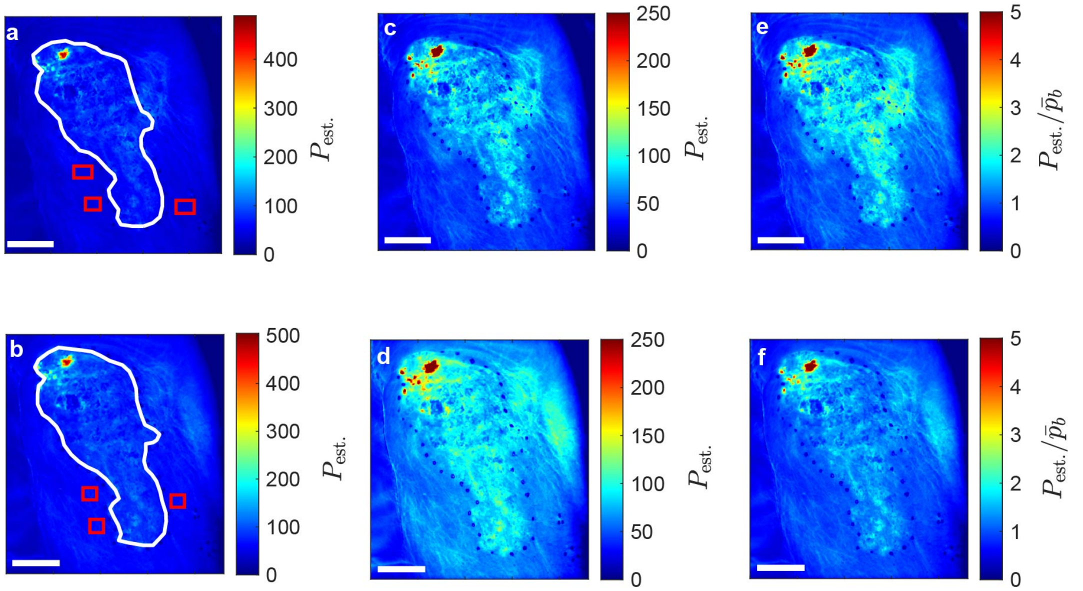

Supplementary Figure S 5. Comparison of mounted (i.e. (a), (c) and (e)) and handheld (i.e. (b), (d) and (f)) measurements. $P_{est.}$: estimated perfusion. Scale bars, 25 mm. Red rectangles: manually selected regions for calculation of background perfusion ($\bar{p}_b$). White polygons: manually selected lesion areas. Temporally averaged perfusion maps with (a-b) a localized color-map scaling and (c-d) the same color-map scaling. (e-f) Corresponding background corrected perfusion maps with the same color-map scaling.

Experiment index 6

	Time	% Aligned frames	$\bar{p}_b$	$\bar{I}$	C_s	C_d
Mounted	13 : 26	100	31.5	15.2	0.78	0.18
Handheld	13 : 24	100	47.8			

Supplementary Table S 6. Detail information of the measurement pair. $\bar{p}_b$: Average perfusion in the selected background areas. $\bar{I}$: Mean intensity on Delrin out of 255. C_s: Speckle contrast on Delrin. C_d: Speckle contrast on scattering suspension.

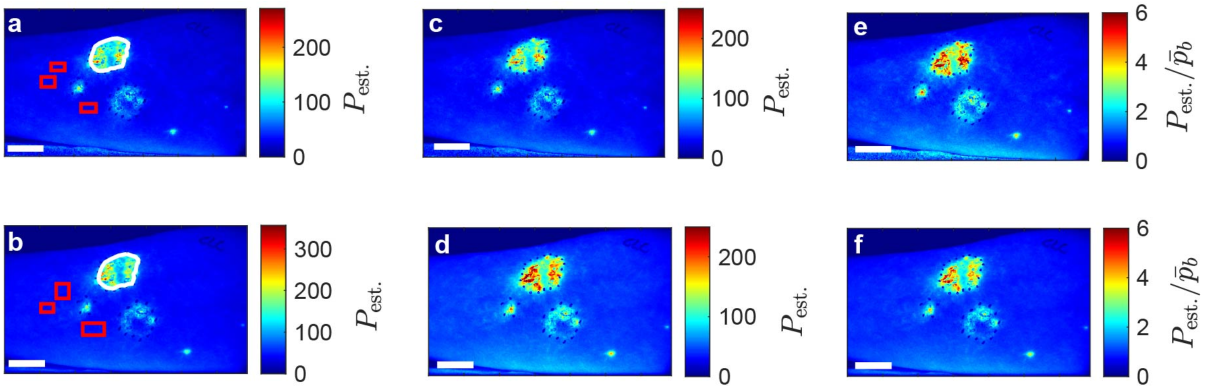

Supplementary Figure S 6. Comparison of mounted (i.e. (a), (c) and (e)) and handheld (i.e. (b), (d) and (f)) measurements. $P_\text{est.}$: estimated perfusion. Scale bars, 25 mm. Red rectangles: manually selected regions for calculation of background perfusion ($\bar{p}_b$). White polygons: manually selected lesion areas. Temporally averaged perfusion maps with (a-b) a localized color-map scaling and (c-d) the same color-map scaling. (e-f) Corresponding background corrected perfusion maps with the same color-map scaling.

Experiment index 7

	Time	% Aligned frames	$\bar{p}_b$	$\bar{I}$	C_s	C_d
Mounted	15 : 39	100	24	20.1	0.94	0.19
Handheld	15 : 37	100	34.4			

Supplementary Table S 7. Detail information of the measurement pair. $\bar{p}_b$: Average perfusion in the selected background areas. $\bar{I}$: Mean intensity on Delrin out of 255. C_s: Speckle contrast on Delrin. C_d: Speckle contrast on scattering suspension.

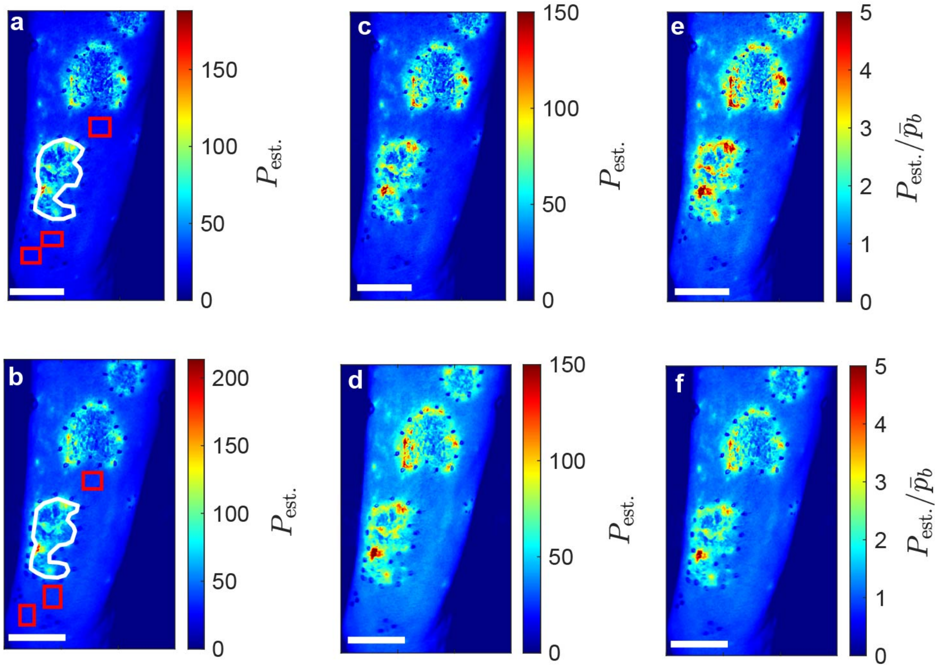

Supplementary Figure S 7. Comparison of mounted (i.e. (a), (c) and (e)) and handheld (i.e. (b), (d) and (f)) measurements. $P_\mathrm{est.}$: estimated perfusion. Scale bars, 25 mm. Red rectangles: manually selected regions for calculation of background perfusion ($\bar{p}_b$). White polygons: manually selected lesion areas. Temporally averaged perfusion maps with (a-b) a localized color-map scaling and (c-d) the same color-map scaling. (e-f) Corresponding background corrected perfusion maps with the same color-map scaling.

Experiment index 8

	Time	% Aligned frames	$\bar{p}_b$	$\bar{I}$	C_s	C_d
Mounted	9 : 26	100	21	20.4	0.9	0.24
Handheld	9 : 23	100	27.2			

Supplementary Table S 8. Detail information of the measurement pair. $\bar{p}_b$: Average perfusion in the selected background areas. $\bar{I}$: Mean intensity on Delrin out of 255. C_s: Speckle contrast on Delrin. C_d: Speckle contrast on scattering suspension.

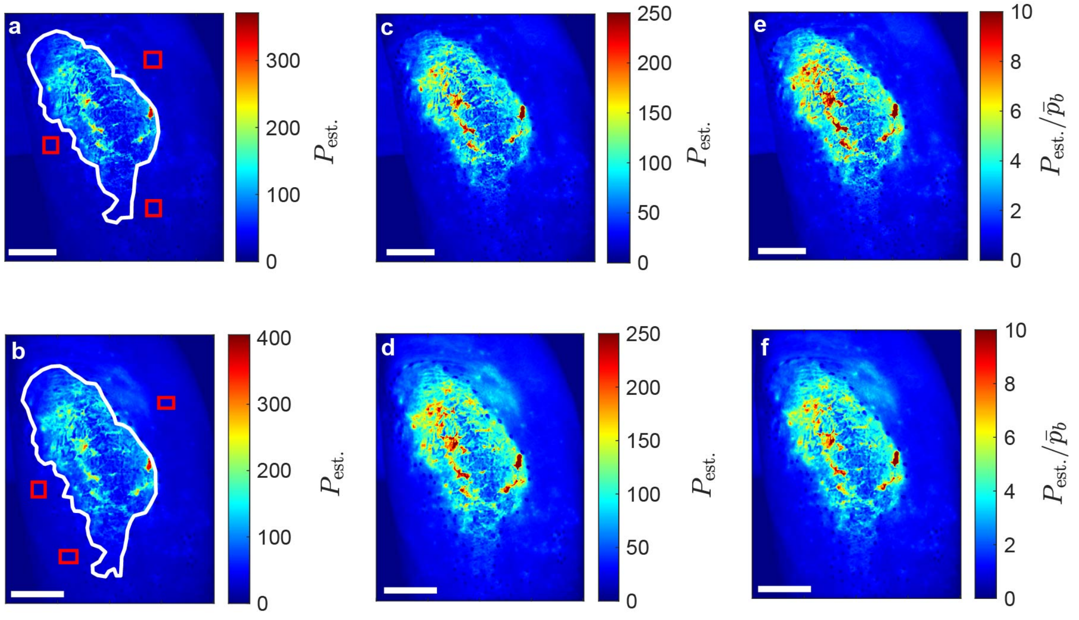

Supplementary Figure S 8. Comparison of mounted (i.e. (a), (c) and (e)) and handheld (i.e. (b), (d) and (f)) measurements. $P_{\mathrm{est.}}$: estimated perfusion. Scale bars, 25 mm. Red rectangles: manually selected regions for calculation of background perfusion ($\bar{p}_b$). White polygons: manually selected lesion areas. Temporally averaged perfusion maps with (a-b) a localized color-map scaling and (c-d) the same color-map scaling. (e-f) Corresponding background corrected perfusion maps with the same color-map scaling.

Experiment index 9

	Time	% Aligned frames	$\bar{p}_b$	$\bar{I}$	C_s	C_d
Mounted	13 : 16	100	37.4	18.1	0.66	0.24
Handheld	13 : 14	58	55			

Supplementary Table S 9. Detail information of the measurement pair. $\bar{p}_b$: Average perfusion in the selected background areas. $\bar{I}$: Mean intensity on Delrin out of 255. C_s: Speckle contrast on Delrin. C_d: Speckle contrast on scattering suspension.

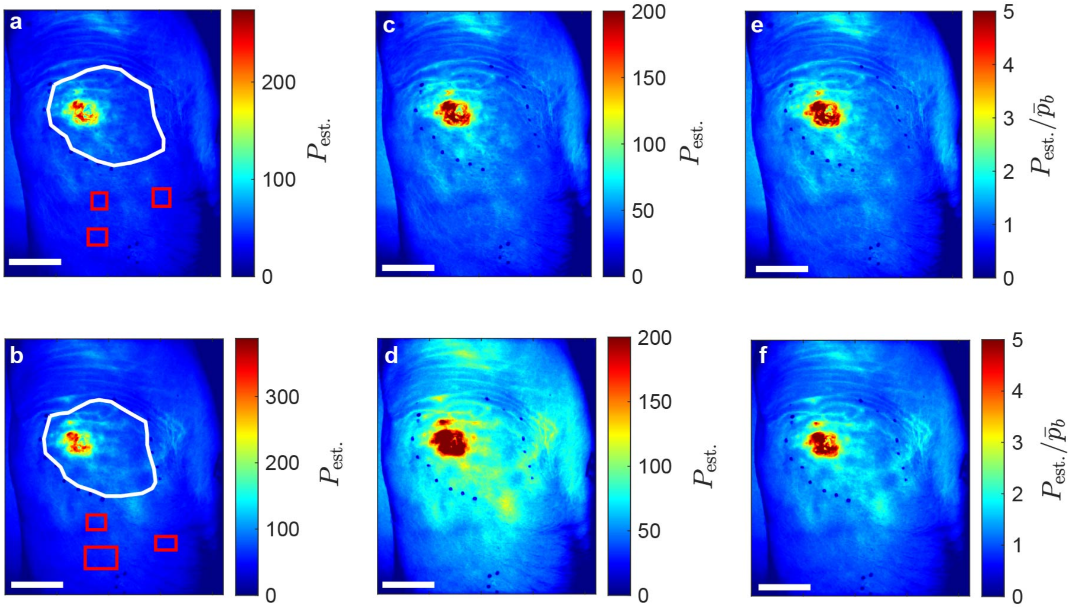

Supplementary Figure S 9. Comparison of mounted (i.e. (a), (c) and (e)) and handheld (i.e. (b), (d) and (f)) measurements. $P_\mathrm{est.}$: estimated perfusion. Scale bars, 25 mm. Red rectangles: manually selected regions for calculation of background perfusion ($\bar{p}_b$). White polygons: manually selected lesion areas. Temporally averaged perfusion maps with (a-b) a localized color-map scaling and (c-d) the same color-map scaling. (e-f) Corresponding background corrected perfusion maps with the same color-map scaling.

Experiment index 10

	Time	% Aligned frames	$\bar{p}_b$	$\bar{I}$	C_s	C_d
Mounted	13 : 25	100	33	15.9	0.64	0.2
Handheld	13 : 23	53	53.5			

Supplementary Table S 10. Detail information of the measurement pair. $\bar{p}_b$: Average perfusion in the selected background areas. $\bar{I}$: Mean intensity on Delrin out of 255. C_s: Speckle contrast on Delrin. C_d: Speckle contrast on scattering suspension.

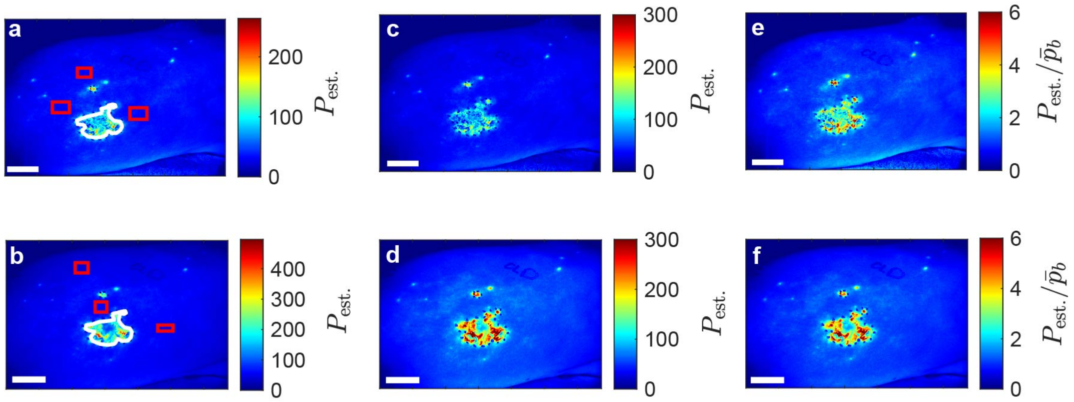

Supplementary Figure S 10. Comparison of mounted (i.e. (a), (c) and (e)) and handheld (i.e. (b), (d) and (f)) measurements. $P_{est.}$: estimated perfusion. Scale bars, 25 mm. Red rectangles: manually selected regions for calculation of background perfusion ($\bar{p}_b$). White polygons: manually selected lesion areas. Temporally averaged perfusion maps with (a-b) a localized color-map scaling and (c-d) the same color-map scaling. (e-f) Corresponding background corrected perfusion maps with the same color-map scaling.

Experiment index 11

	Time	% Aligned frames	$\bar{p}_b$	$\bar{I}$	C_s	C_d
Mounted	14 : 24	100	25.5	20	0.85	0.22
Handheld	14 : 22	57	36.5			

Supplementary Table S 11. Detail information of the measurement pair. $\bar{p}_b$: Average perfusion in the selected background areas. $\bar{I}$: Mean intensity on Delrin out of 255. C_s: Speckle contrast on Delrin. C_d: Speckle contrast on scattering suspension.

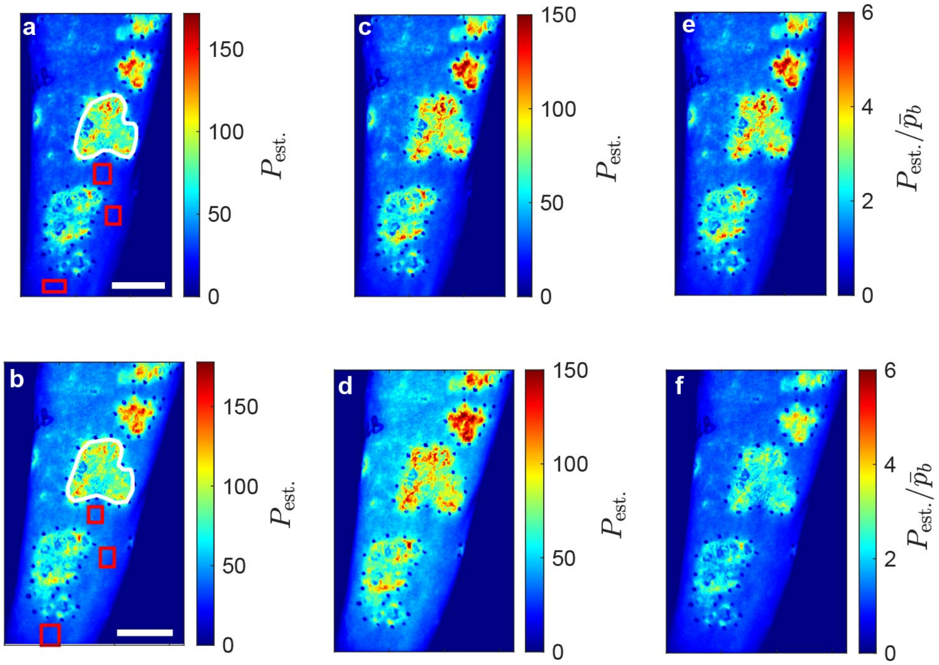

Supplementary Figure S 11. Comparison of mounted (i.e. (a), (c) and (e)) and handheld (i.e. (b), (d) and (f)) measurements. $P_{est.}$: estimated perfusion. Scale bars, 25 mm. Red rectangles: manually selected regions for calculation of background perfusion ($\bar{p}_b$). White polygons: manually selected lesion areas. Temporally averaged perfusion maps with (a-b) a localized color-map scaling and (c-d) the same color-map scaling. (e-f) Corresponding background corrected perfusion maps with the same color-map scaling.
