# Supplementary material for: Handheld versus mounted laser speckle contrast perfusion imaging demonstrated in psoriasis lesions
Source: Sci Rep. 2021 Aug 17;11:16646. doi: 10.1038/s41598-021-96218-6 (PMC8371022; doi:10.1038/s41598-021-96218-6)
Supplement: Supplementary file 2 — Supplementary Code S1. [file 41598_2021_96218_MOESM2_ESM.zip › Supplementary Code S 1/code for processing raw data/1 segmentation and alignment/seg_align_App_report.html]

Migration Report for <code>seg\_align</code>


# Migration Report for `seg_align`

### Your GUIDE app was migrated to App Designer and some action is required.

## Migration Results

- **seg\_align\_App.mlapp** file created
- **395** lines of code analyzed in seg\_align.m
- **18** components created and initialized
- **11** callbacks and utility functions configured and enabled to function in App Designer

## Issues Requiring Action

| Functionality | GUIDE Tag / Line # | Details |
| --- | --- | --- |
| `WindowStyle` | `figure1` | This property for the component(s) is not supported in App Designer and was not migrated.  Action:Determine if this functionality is critical to your app before continuing because there is no workaround. |
| `clf` | line 182 | This function is not supported in App Designer code.  Action:Determine if this functionality is critical to your app before continuing because there is no workaround. |

## Validate Your Migrated App

The migration tool enables most of your app code to execute. Now, you need to fully validate your migrated app to make sure it behaves as expected. Consider doing the following verifications:

- Verify Layout
  - Run your app and verify the initial layout of all components is as expected.
  - If your app dynamically shows, hides, or enables additional components or options in response to selections made within the app, confirm these behave as expected.
- Verify Callbacks
  - Run and close your app to confirm there are no startup or termination errors.
  - Exercise the workflows specific to your app, starting with the most basic, and gradually proceeding to the more complex ones.

If unexpected errors occur, either within the app or at the MATLAB command line, address those issues before continuing with the validation. For troubleshooting assistance and additional information, see GUIDE Migration Strategies.
