# Supplementary figures and images for: Handheld versus mounted laser speckle contrast perfusion imaging demonstrated in psoriasis lesions
Source: Sci Rep. 2021 Aug 17;11:16646. doi: 10.1038/s41598-021-96218-6 (PMC8371022; doi:10.1038/s41598-021-96218-6)

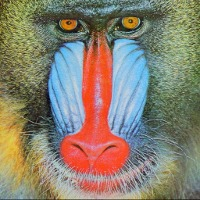

Supplement: Supplementary file 2 — Supplementary Code S1. [file 41598_2021_96218_MOESM2_ESM.zip › Supplementary Code S 1/code for processing raw data/1 segmentation and alignment/IAT_v0.9.3/IAT_v0.9.3/data/BaboonImage.png]

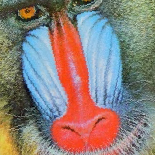

Supplement: Supplementary file 2 — Supplementary Code S1. [file 41598_2021_96218_MOESM2_ESM.zip › Supplementary Code S 1/code for processing raw data/1 segmentation and alignment/IAT_v0.9.3/IAT_v0.9.3/data/BaboonTemplate.png]

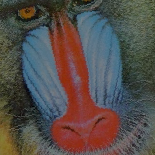

Supplement: Supplementary file 2 — Supplementary Code S1. [file 41598_2021_96218_MOESM2_ESM.zip › Supplementary Code S 1/code for processing raw data/1 segmentation and alignment/IAT_v0.9.3/IAT_v0.9.3/data/BaboonTemplateIntensityChange.png]

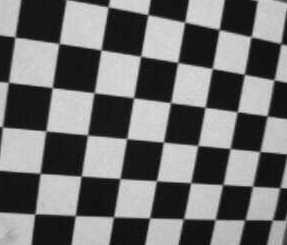

Supplement: Supplementary file 2 — Supplementary Code S1. [file 41598_2021_96218_MOESM2_ESM.zip › Supplementary Code S 1/code for processing raw data/1 segmentation and alignment/IAT_v0.9.3/IAT_v0.9.3/data/chessImage.png]

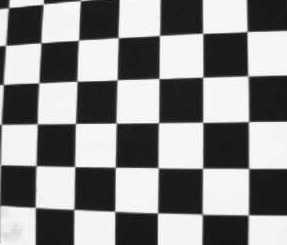

Supplement: Supplementary file 2 — Supplementary Code S1. [file 41598_2021_96218_MOESM2_ESM.zip › Supplementary Code S 1/code for processing raw data/1 segmentation and alignment/IAT_v0.9.3/IAT_v0.9.3/data/chessTemplate.png]

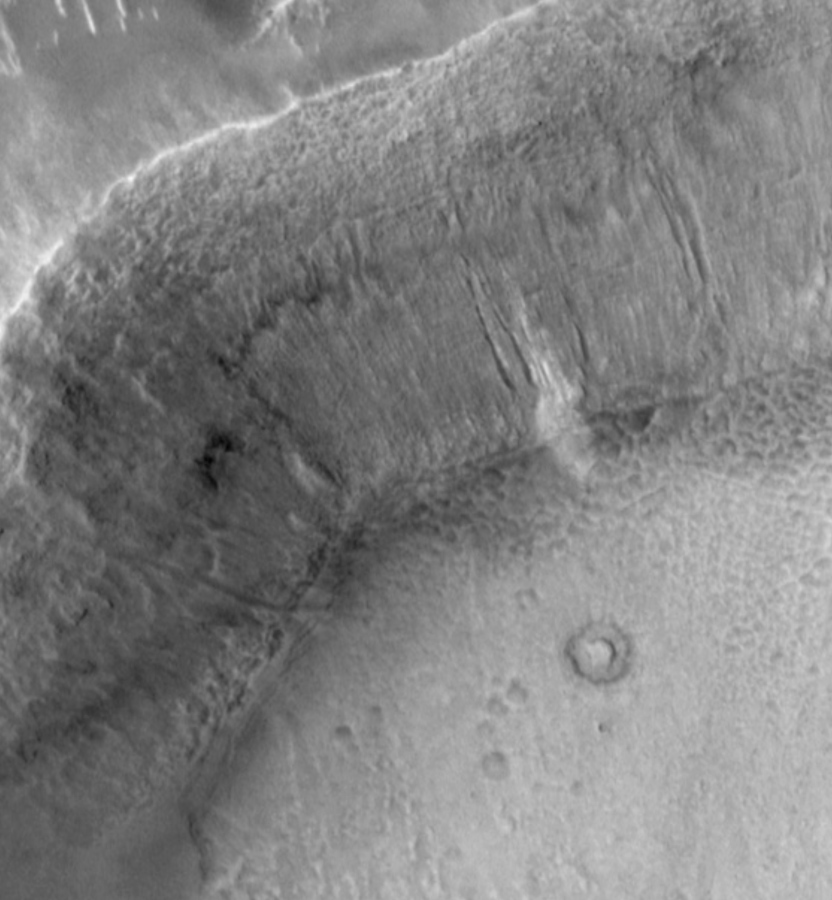

Supplement: Supplementary file 2 — Supplementary Code S1. [file 41598_2021_96218_MOESM2_ESM.zip › Supplementary Code S 1/code for processing raw data/1 segmentation and alignment/IAT_v0.9.3/IAT_v0.9.3/data/Mars1.png]

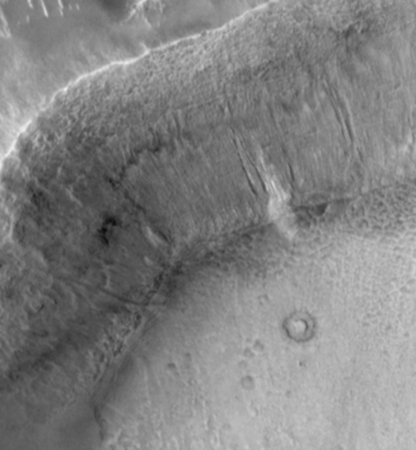

Supplement: Supplementary file 2 — Supplementary Code S1. [file 41598_2021_96218_MOESM2_ESM.zip › Supplementary Code S 1/code for processing raw data/1 segmentation and alignment/IAT_v0.9.3/IAT_v0.9.3/data/Mars1HalfSize.png]

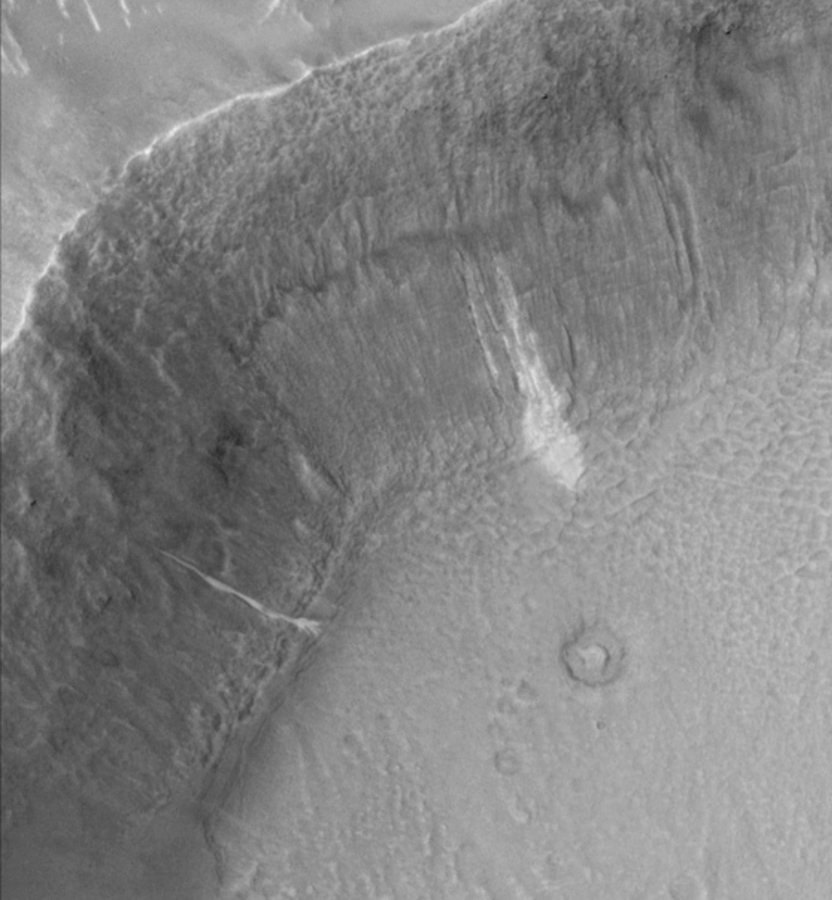

Supplement: Supplementary file 2 — Supplementary Code S1. [file 41598_2021_96218_MOESM2_ESM.zip › Supplementary Code S 1/code for processing raw data/1 segmentation and alignment/IAT_v0.9.3/IAT_v0.9.3/data/Mars2.png]

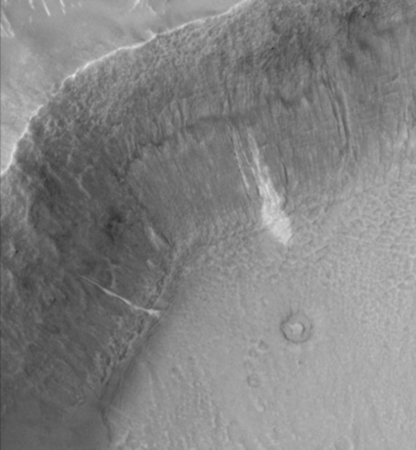

Supplement: Supplementary file 2 — Supplementary Code S1. [file 41598_2021_96218_MOESM2_ESM.zip › Supplementary Code S 1/code for processing raw data/1 segmentation and alignment/IAT_v0.9.3/IAT_v0.9.3/data/Mars2HalfSize.png]

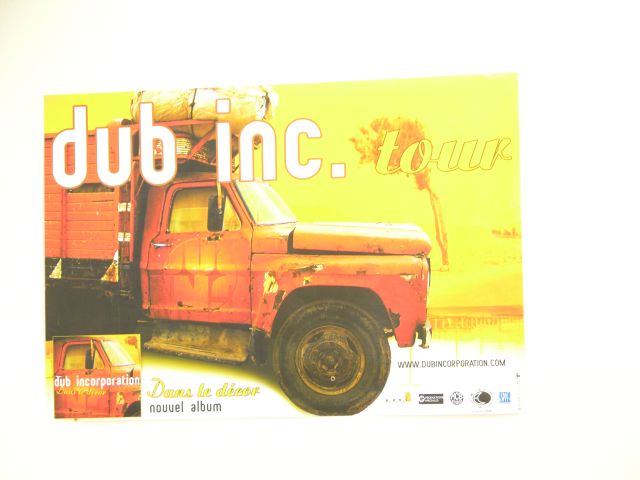

Supplement: Supplementary file 2 — Supplementary Code S1. [file 41598_2021_96218_MOESM2_ESM.zip › Supplementary Code S 1/code for processing raw data/1 segmentation and alignment/IAT_v0.9.3/IAT_v0.9.3/data/source.jpg]

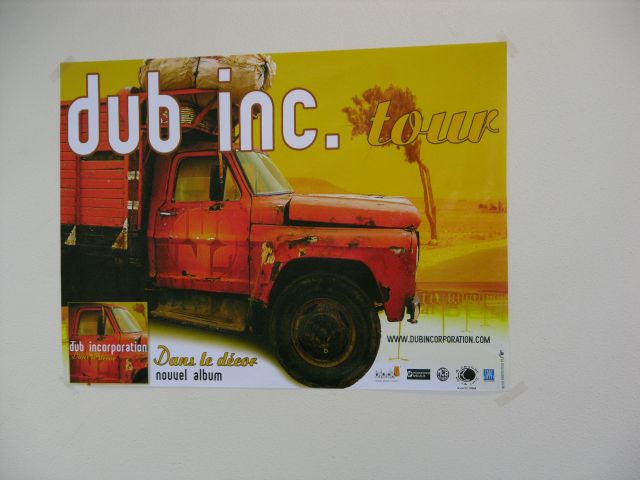

Supplement: Supplementary file 2 — Supplementary Code S1. [file 41598_2021_96218_MOESM2_ESM.zip › Supplementary Code S 1/code for processing raw data/1 segmentation and alignment/IAT_v0.9.3/IAT_v0.9.3/data/target.jpg]

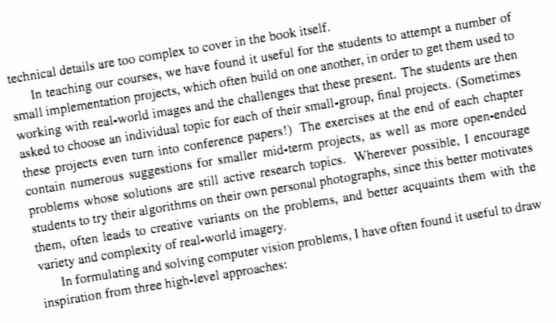

Supplement: Supplementary file 2 — Supplementary Code S1. [file 41598_2021_96218_MOESM2_ESM.zip › Supplementary Code S 1/code for processing raw data/1 segmentation and alignment/IAT_v0.9.3/IAT_v0.9.3/data/textImage.png]

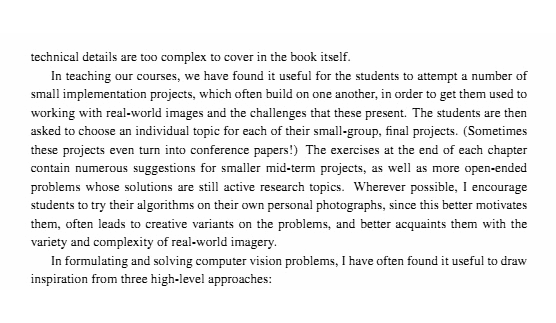

Supplement: Supplementary file 2 — Supplementary Code S1. [file 41598_2021_96218_MOESM2_ESM.zip › Supplementary Code S 1/code for processing raw data/1 segmentation and alignment/IAT_v0.9.3/IAT_v0.9.3/data/textTemplate.png]

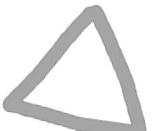

Supplement: Supplementary file 2 — Supplementary Code S1. [file 41598_2021_96218_MOESM2_ESM.zip › Supplementary Code S 1/code for processing raw data/1 segmentation and alignment/IAT_v0.9.3/IAT_v0.9.3/data/triangleImage.png]

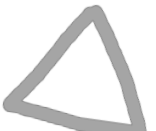

Supplement: Supplementary file 2 — Supplementary Code S1. [file 41598_2021_96218_MOESM2_ESM.zip › Supplementary Code S 1/code for processing raw data/1 segmentation and alignment/IAT_v0.9.3/IAT_v0.9.3/data/triangleImage2.png]

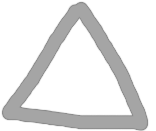

Supplement: Supplementary file 2 — Supplementary Code S1. [file 41598_2021_96218_MOESM2_ESM.zip › Supplementary Code S 1/code for processing raw data/1 segmentation and alignment/IAT_v0.9.3/IAT_v0.9.3/data/triangleTemplate.png]

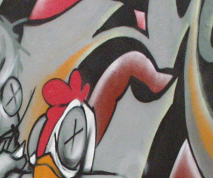

Supplement: Supplementary file 2 — Supplementary Code S1. [file 41598_2021_96218_MOESM2_ESM.zip › Supplementary Code S 1/code for processing raw data/1 segmentation and alignment/IAT_v0.9.3/IAT_v0.9.3/data/wallImage.png]

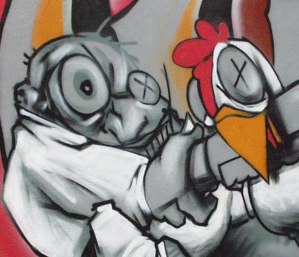

Supplement: Supplementary file 2 — Supplementary Code S1. [file 41598_2021_96218_MOESM2_ESM.zip › Supplementary Code S 1/code for processing raw data/1 segmentation and alignment/IAT_v0.9.3/IAT_v0.9.3/data/wallTemplate.png]

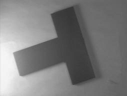

Supplement: Supplementary file 2 — Supplementary Code S1. [file 41598_2021_96218_MOESM2_ESM.zip › Supplementary Code S 1/code for processing raw data/1 segmentation and alignment/LocalizedActiveContour/1.bmp]

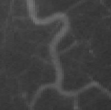

Supplement: Supplementary file 2 — Supplementary Code S1. [file 41598_2021_96218_MOESM2_ESM.zip › Supplementary Code S 1/code for processing raw data/1 segmentation and alignment/LocalizedActiveContour/2.bmp]

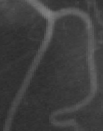

Supplement: Supplementary file 2 — Supplementary Code S1. [file 41598_2021_96218_MOESM2_ESM.zip › Supplementary Code S 1/code for processing raw data/1 segmentation and alignment/LocalizedActiveContour/3.bmp]

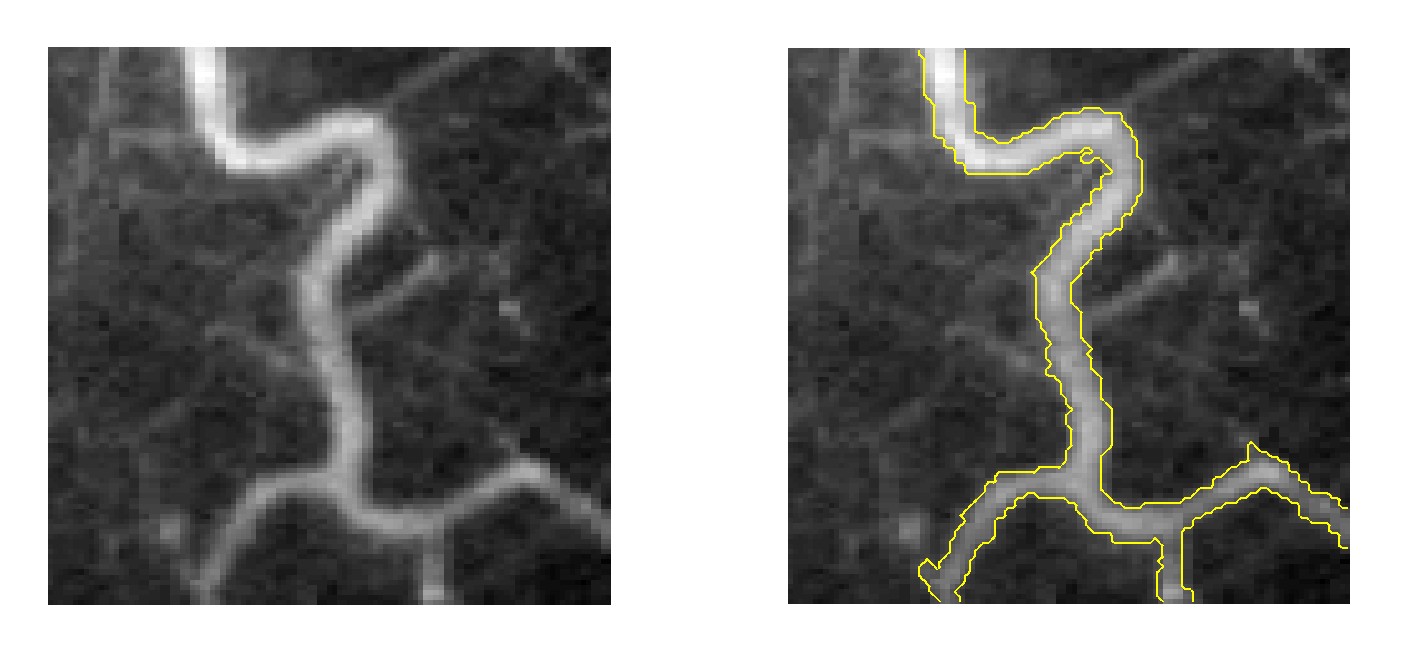

Supplement: Supplementary file 2 — Supplementary Code S1. [file 41598_2021_96218_MOESM2_ESM.zip › Supplementary Code S 1/code for processing raw data/1 segmentation and alignment/LocalizedActiveContour/results.png]
